# Comparison of host genetic factors influencing pig response to infection with two North American isolates of porcine reproductive and respiratory syndrome virus

**DOI:** 10.1186/s12711-016-0222-0

**Published:** 2016-06-20

**Authors:** Andrew S. Hess, Zeenath Islam, Melanie K. Hess, Raymond R. R. Rowland, Joan K. Lunney, Andrea Doeschl-Wilson, Graham S. Plastow, Jack C. M. Dekkers

**Affiliations:** Department of Animal Science, Iowa State University, Ames, IA USA; The Roslin Institute and R(D)SVS, University of Edinburgh, Edinburgh, Midlothian UK; College of Veterinary Medicine, Kansas State University, Manhattan, KS USA; USDA, ARS, BARC, Beltsville, MD USA; University of Alberta, Edmonton, AB Canada

## Abstract

**Background:**

Porcine reproductive and respiratory syndrome (PRRS) is one of the most important swine diseases in the world and genetic selection of pigs for increased resistance to PRRS is an attractive method to improve the health status of the swine herd. This study compared phenotypic and genetic responses to infection with one of two genetically distinct type 2 PRRS virus (PRRSV) isolates: NVSL-97-7895 (NVSL) and KS-2006-72109 (KS06), and evaluated whether the single nucleotide polymorphism (SNP) WUR10000125 (WUR) on chromosome 4 that was associated with viral load and weight gain under infection with NVSL also has an effect on response to infection across North American PRRSV isolates. Wood’s lactation curve was fitted to repeated viremia measurements to derive five curve characteristics that were evaluated.

**Results:**

Infection with NVSL was characterized by reaching a 14 ± 2 % higher peak viremia (PV) 2.5 ± 0.6 days earlier (time to peak; TP) than KS06, followed by 36 ± 1 % faster virus clearance, which occurred 3.9 ± 0.7 days sooner. Weight gain from 0 to 42 days post-infection (WG) tended to be higher under infection with KS06 than NVSL (3.7 ± 1.5 kg). Estimates of heritability were moderate for both PRRSV isolates for viral load from 0 to 21 days post-infection (VL) (NVSL: 0.31 ± 0.06; KS06: 0.51 ± 0.09) and WG (NVSL: 0.33 ± 0.06; KS06: 0.31 ± 0.09). Strong negative genetic correlations were observed between VL and WG for both NVSL (−0.74 ± 0.10) and KS06 (−0.52 ± 0.17) infected pigs. Pigs with genotype AB at the WUR SNP had a more desirable phenotype than AA pigs for all traits under infection with NVSL, but only for VL and PV with KS06; effects on other traits were smaller and not significantly different from zero (P > 0.05). Genetic correlations of host response between isolates were strong for VL, WG and PV. Accounting for WUR genotype had little impact on these correlations, suggesting that response to PRRSV infection has a substantial polygenic component that is common between these two isolates.

**Conclusions:**

These results suggest that the KS06 PRRSV isolate is less virulent than NVSL but that genetic selection for increased resistance to either of these genetically distinct isolates is expected to increase resistance to the other isolate.

**Electronic supplementary material:**

The online version of this article (doi:10.1186/s12711-016-0222-0) contains supplementary material, which is available to authorized users.

## Background

Porcine reproductive and respiratory syndrome (PRRS) costs the US swine industry $664 million per year [1]. Past efforts to contain PRRS have had limited success, in large part due to the high mutation rate and antigenic variability of PRRS virus (PRRSV), which have encumbered efforts to produce vaccines that are cross-protective to heterogeneous PRRSV isolates [2]. PRRSV isolates are classified into two types: type 1 or European isolates and type 2 or North American isolates [3]. These two types are distinct both genetically [4] and pathogenically [5, 6].

Genetic selection of pigs that are more resistant to PRRS can be an attractive method to improve the health status of the swine herd [7]. The goal of the PRRS Host Genetics Consortium (PHGC) is to identify host genes or genomic regions that are associated with increased resistance of pigs to PRRSV infection [8]. Previous studies using multiple contemporary North American crossbred weaner pigs that were experimentally infected with a North American isolate of PRRSV, NVSL-97-7895 (NVSL), identified heritable genetic components to viral load and weight gain following infection, and identified a quantitative trait locus (QTL) on chromosome 4 whereby the single nucleotide polymorphism (SNP) WUR10000125 (WUR) had a strong association with these two host response traits [9–11]. A putative causative mutation that is in high linkage disequilibrium with the WUR SNP, in the *guanylate binding protein 5* (*GBP5*) gene, was identified by Koltes et al. [12]. The protein produced by the *GBP5* gene plays a crucial role in NLRP3-mediated formation of the inflammasome, which is involved with inflammatory response [13].

It is currently not known whether selection for improved host resistance to a single PRRSV isolate will improve resistance to other PRSSV isolates. Thus, the objectives of this study were to: (1) compare host responses to infection with NVSL and the genetically distinct North American PRRSV isolate KS-2006-72109 (KS06); (2) estimate the genetic parameters of response to infection when pigs are infected with either NVSL or KS06; and (3) estimate the associations of the WUR SNP with response following infection with NVSL or KS06. It was hypothesized that the host’s genetic make-up that is involved in the response to infection is highly correlated between these two virus isolates and that associations of the WUR SNP with host response to infection are similar for these two isolates. The Wood’s lactation function was previously shown to appropriately model PRRS serum viremia following experimental infection [14]. Thus, curve characteristics of the fitted viremia profiles that are derived from the Wood’s curve parameters were used to quantify different aspects of the dynamics of host response to PRRSV infection with these two isolates.

## Methods

### Study design

A detailed description of the design, data collection and molecular techniques used in the PHGC trials is in Lunney et al. [8]. The Kansas State University Institutional Animal Care and Use Committee approved all experimental protocols for these trials. Pigs used for this study were from 14 PHGC trials of ~200 weaner pigs (Table 1). Pigs were provided from commercial breeding programs in the United States and Canada. Within each trial, pigs were from a single high health farm and genetic background, except for trials 5, 8 and 12, which each included pigs from one genetic background but from two farms. All source farms were free of PRRS, *Mycoplasma hyopneumoniae*, and swine influenza. Four breeding companies supplied pigs of the same breed cross for more than one trial, with pigs in one trial infected with KS06 and in one or more trials with NVSL (Table 1). Pigs from the same breeding company and the same breed cross were from the same genetic background.

**Table 1:** Animal composition of the PHGC trials

PRRS virus isolate	Trial #	Number of animals	Number of sires	Number of dams	Breed cross	Genetic background^a^
NVSL-97-7895	1–3	507	32	203	LW × LR	A
	4	191	6	33	Duroc × LW/LR	B
	5	182	10	38	Duroc × LR/LW	C
	6	109	26	53	LR × LR	D
	7	186	6	27	Pietrain × LW/LR	E
	8	158	15	43	Duroc × LW/LR	F
	15	166	11	49	Pietrain × LW	G
KS-2006-72109	10	184	8	57	Pietrain × LW	G
	11	177	37	89	LW × LR	A
	12	146	14	49	LR × LW	H
	13^b^	173	20	37	Duroc × LW/LR	F
	14	165	8	24	Duroc × LR/LW	C

*LW* large white, *LR* landrace

^a^Genetic background is defined as pigs from the same breeding company and the same breed cross

^b^Trial 13 was excluded from analyses due to unusual viremia profiles as seen in Additional file 1: Figure S1

For each trial, animals were transported to Kansas State University at weaning (average age of 21 days) and randomly placed into pens of 10 to 15 pigs. After a 7-day acclimation period, pigs were experimentally infected, both intramuscularly and intranasally, with 10^5^ TCID50 of NVSL-97-7985, a highly virulent type 2 PRRSV isolate [15], for trials 1 to 8 and 15 and 10^5^ TCID50 of KS-2006-72109, a more contemporary type 2 PRRSV isolate, for trials 10 to 14. NVSL and KS06 were isolated from different geographic regions nearly ten years apart, share a 89 % similarity at both the glycoprotein 5 (*GP5*) nucleotide and amino acid sequence levels [16, 17] and are located in two distinct molecular phylogenetic branches of PRRSV [16]. The *GP5* gene is often used to assess genetic differences between PRRSV isolates and is suggestive of differences in virulence between isolates [18, 19]. Forsberg et al. [20] found that, on average, PRRSV isolates have a substitution rate of 0.073 per nucleotide in *GP5*, with the maximum substitution rate between two isolates being 0.153; NVSL and KS06 have a substitution rate of 0.11 per nucleotide in the *GP5* gene [17].

Blood samples were collected at −6, 0, 4, 7, 11, 14, 21, 28, 35 and 42 days post-infection (dpi). Body weight was measured at 0, 7, 14, 21, 28, 35 and 42 dpi. Pigs were euthanized at 42 dpi. Trials 7 and 8 were stopped at 35 dpi due to unavailability of the facility. Serum viremia was measured using a semi-quantitative TaqMan polymerase chain reaction assay for PRRSV ribonucleic acid (RNA), as described in Boddicker et al. [9–11] and Ladinig et al. [16]. Assay results were reported as the log_10_ of PRRSV RNA copies per mL of serum. A time course of viremia levels for each animal within a trial was plotted in order to provide an initial assessment of response to infection and to confirm that all animals were infected (see Additional file 1: Figure S1). Trial 13 was excluded from further analyses due to unusual viremia profiles that were not observed in any other PHGC trial; some trial 13 animals showed delayed presence of serum viremia and all pigs had low and highly variable viremia levels over time compared to the other trials, which suggested that the virus was attenuated or the pigs were not naïve. The latter could be due to the presence of maternal antibodies resulting from a previous infection or vaccination in the source herd [21].

Across all nine trials infected with NVSL, 12 % of pigs died or were euthanized for humane reasons before 42 dpi. Mortality rate was similar in the five KS06 trials, with 9 % pigs dying or euthanized before 42 dpi. Dead pigs were necropsied and subsequent gross and microscopic pathology by a board-certified pathologist identified PRRS associated disease as the major source of mortality, except for trial 6. Mortality rate was high in trial 6 (46 % by day 42), due to secondary bacterial infections, as identified by pathology. Secondary bacterial infections included *Escherichia coli*, *Streptococcus suis*, *Staphylococcus aureus*, and *Mycoplasma hyopneumoniae* [7], which were subsequently traced to the pig supplier. The impact of this co-infection on pig performance was further investigated by Boddicker et al. [22]. The animals from all other trials remained negative of secondary infections.

### Genotyping and pedigree

Ear tissue was collected from all pigs for DNA isolation. DNA samples from trials 1 to 10 were genotyped with Illumina’s Porcine SNP60 Beadchip [23] v1 (San Diego, California) at GeneSeek Inc. (Lincoln, Nebraska) and samples from trials 11 to 15 were genotyped with Illumina’s Porcine SNP60 Beadchip v2 (San Diego, California) at Delta Genomics (Edmonton, Alberta). Only SNPs that were on both versions of the Illumina’s Porcine SNP60 Beadchip were used in this study. SNPs were removed if they were fixed within a trial or if they were unmapped or mapped to a sex chromosome in build 10.2 of the swine genome (GenBank Accession: GCA_000003025.4); this left 48,164 SNPs. No additional filters were applied to the animals or genotypes. The animal with the smallest number of called SNPs had a call rate of 0.82 (the 99th percentile was 0.98), while the SNP with the lowest call rate among animals had a call rate of 0.62 (99th percentile 0.97). Missing genotypes were assigned the average genotype (on a 0, 1, 2 scale) for animals in that trial for that SNP. This set of SNPs will be referred to as 60k SNPs.

Pedigree information was available for all pigs in all trials. Trials 1, 2 and 3, which consisted of animals from the same breeding company in consecutive parities, had the most extensive pedigree information, with records up to two generations back, while only sire and dam information was available for the other trials. As such, there were no relationships between animals in different trials, except for trials 1, 2, and 3. Pedigree was corrected using parental genotypes for trials 1 through 8, as described by Boddicker et al. [11]. The 1250 highest quality 60k SNPs, based on GC score and call rate, were used in Cervus 3.0 [15] to verify pedigree information for trials 11 and 15, and assign sires for trials 12 and 13, which used pooled semen [24]. Parental genotypes were not available for trials 10 and 14 and the pedigree provided was assumed to be correct.

### Viremia curve characteristics

In previous studies, Boddicker et al. [9–11] used viral load, defined as area under the curve of log_10_ viral copies/ml of serum from 0 to 21 dpi, as a measurement of response to PRRSV infection. Area under the curve is a summary phenotype of the viral burden but it does not explicitly capture the dynamics of an individual animal’s curve that can influence this viral burden; two animals that have different viremia curves can have the same viral load. Analysis of different aspects of the viremia curve may aid in understanding differences in virulence of the two virus isolates, as well as provide insight into the role that the host’s genetics plays in response to infection [14]. The genetic mechanisms for one curve characteristic may be conserved across isolates, while another curve characteristic may be variable across isolates.

The Wood’s curve, an incomplete gamma function often used to model lactation yield in dairy cattle [25–27], was shown to appropriately model viremia profiles in PHGC trials 1–8 [14]:

$${\text{V}}\left( {\text{t}} \right) = {\text{a}}_{1} {\text{t}}^{{{\text{b}}_{1} }} {\text{e}}^{{ - {\text{c}}_{1} {\text{t}}}} ,$$

where V(t) is serum viremia on the log_10_ scale at t dpi, ${\text{a}}_{1}$ is a parameter that impacts the magnitude of all points on the curve, ${\text{b}}_{1}$ is an indicator of the initial rate of increase to peak viremia, and ${\text{c}}_{1}$ is an indicator of the rate of decline after the peak and dominates the viremia profile at later stages of infection. These three function parameters were estimated for each individual that had measurements for at least five time points, using Bayesian inference with a likelihood framework, implemented by a Markov chain Monte Carlo method, as described in Islam et al. [14].

The raw viremia profiles of some pigs appeared bi-modal, so an extended Wood’s curve was also fitted for each piglet using the same methodology [14]:

$${\text{V}}\left( {\text{t}} \right) = {\text{a}}_{1} {\text{t}}^{{{\text{b}}_{1} }} {\text{e}}^{{ - {\text{c}}_{1} {\text{t}}}} + { \hbox{max} }\left( {0,{\text{a}}_{2} \left( {{\text{t}} - {\text{t}}_{0} } \right)^{{{\text{b}}_{2} }} {\text{e}}^{{ - {\text{c}}_{2} \left( {{\text{t}} - {\text{t}}_{0} } \right)}} } \right),$$

where ${\text{t}}_{0}$ denotes the time of onset of the second phase of the profile, which is assumed to follow the same Wood’s shape as the primary phase and is thus defined by a second set of Wood’s model parameters. A piglet was classified as experiencing viremia rebound based on the Akaike’s Information Criterion (AIC) if AIC_WOOD’S_-AIC_EXTENDEDWOOD’S_ was greater than 1.488, which corresponds to the 95 % significance level of the likelihood ratio test between these models [14]. For a summary of estimates of the curve parameters $(\widehat{{{\text{a}}_{1} }},\widehat{{{\text{b}}_{1} }},\widehat{{{\text{c}}_{1} }},\widehat{{{\text{a}}_{2} }},\widehat{{{\text{b}}_{2} }},\widehat{{{\text{c}}_{2} }})$ and the proportion of animals classified as having a cleared, rebound, or persistent serum viremia profile, see Additional file 2: Table S1. A more detailed description of the fitting of the Wood’s and Extended Wood’s curve, including a visualization of the curves, is provided in Islam et al. [14].

Using the estimates of the curve parameters ($\widehat{{{\text{a}}_{1} }},\widehat{{{\text{b}}_{1} }},\widehat{{{\text{c}}_{1} }}$) of the single or the extended Wood’s curve for each pig, five curve characteristics were derived to describe the viremia profile of each pig. For pigs with extended Wood’s curves, only estimates of parameters of the primary phase were used in this study because this phase was previously shown to have a heritable genetic component, while heritability for rebound was previously estimated to be low (0.03), suggesting that rebound is largely governed either by viral escape or other environmental factors [14].

The first characteristic evaluated, area under the Wood’s curve, hereafter referred to as viral load (VL), was given by the definite integral for each individual (i):

$${\text{VL}}_{\text{i}} = \mathop \int \nolimits_{0}^{21} \widehat{{{\text{a}}_{{1{\text{i}}}} }}{\text{t}}^{{\widehat{{{\text{b}}_{{1{\text{i}}}} }}}} {\text{e}}^{{ - \widehat{{{\text{c}}_{{1{\text{i}}}} }}{\text{t}}}}\,{\text{dt}} .$$

VL is a measure of both the level of viremia and the extent to which viremia is maintained. The range 0–21 dpi was chosen to capture the uni-modal phase of infection common to all pigs. Previous analyses of viremia from trials 1 to 8 fitted a LOESS curve through viremia and integrated to obtain area under the curve from 0 to 21 dpi [9–11]. This measure is denoted by ${\text{VL}}_{\text{B}}$. Since it was not known how similar ${\text{VL}}_{\text{B}}$ and VL were, which may impact interpretation and comparisons with previous studies, a bivariate model using pedigree information was fitted to the VL and ${\text{VL}}_{\text{B}}$ data, separately for the KS06 and NVSL trials, using ASReml 3.0 [28]. Based on similar heritabilities and high genetic and phenotypic correlations between VL and ${\text{VL}}_{\text{B}}$ for both isolates, it was concluded that VL based on the Wood’s curve describes the same biological trait as ${\text{VL}}_{\text{B}}$ (Table 2). Therefore, VL derived from the Wood’s curve was used for all remaining analyses.

**Table 2:** Comparison of viral load of Boddicker (VL_B_) and viral load based on the Wood’s curve (VL) for the two virus isolates (NVSL and KS06)

VL_B_ versus VL^a^	Heritability	Genetic correlation	Phenotypic correlation
NVSL VL_B_	0.23 (0.10)	0.98 (0.03)	0.90 (0.01)
NVSL VL	0.22 (0.10)		
KS06 VL_B_	0.35 (0.09)	0.98 (0.02)	0.90 (0.01)
KS06 VL	0.35 (0.09)		

Data from NVSL and KS06 infection trials were analyzed separately

^a^All trials except trial 13 were used in the analysis

The second curve characteristic evaluated was time (in dpi) to peak viremia (TP), derived by setting the first derivative of the Wood’s equation to zero and solving for t, resulting in:

$${\text{TP}}_{i} = \frac{{\widehat{{{\text{b}}_{1i} }}}}{{\widehat{{{\text{c}}_{1i} }}}}.$$

The third curve characteristic was peak viremia (PV), which was calculated by setting ${\text{t }} = {\text{TP}}$ in the expression for the Wood’s curve:

$${\text{PV}}_{i} = \widehat{{{\text{a}}_{1i} }}\left( {\frac{{\widehat{{{\text{b}}_{1i} }}}}{{\widehat{{{\text{c}}_{1i} }}}}} \right)^{{\widehat{{{\text{b}}_{1i} }}}} {\text{e}}^{{ - \widehat{{{\text{b}}_{1i} }}}} .$$

TP and PV are related to the host’s ability to respond during the replication-dominant phase of early PRRSV infection [29]. PV is reached when the rate of virus clearance from serum is equal to the number of virus particles released into the blood stream. TP is the time it takes to reach PV, with the animals that can mount a response early in infection expected to have a shorter TP.

Curve characteristics that relate to the host’s response during the post-peak, clearance-dominant phase of PRRSV infection were also evaluated. The maximal decay rate (${\text{Vmax}}$) is reached when the rate of viral clearance from serum is highest compared to the rate of viral replication. Time to maximal decay (${\text{Tmax}}$) was derived by setting the second derivative of the Wood’s equation to zero and solving for t:

$${\text{Tmax}}_{i} = \frac{{\widehat{{{\text{b}}_{1i} }} + \sqrt {\widehat{{{\text{b}}_{1i} }}} }}{{\widehat{{{\text{c}}_{1i} }}}}.$$

Substituting this value for t in the first derivative and taking the absolute value gives ${\text{Vmax}}$:

$${\text{Vmax}}_{i} = \left| { - \widehat{{{\text{a}}_{1i} }}\sqrt {\widehat{{{\text{b}}_{1i} }}} \left( {\frac{{\widehat{{{\text{b}}_{1i} }} + \sqrt {\widehat{{{\text{b}}_{1i} }}} }}{{\widehat{{{\text{c}}_{1i} }}}}} \right)^{{\widehat{{{\text{b}}_{1i} }} - 1}} {\text{e}}^{{ - \left( {\widehat{{{\text{b}}_{1i} }} + \sqrt {\widehat{{{\text{b}}_{1i} }}} } \right)}} } \right|.$$

${\text{Vmax}}$ was defined as the absolute value, such that for an animal with a larger ${\text{Vmax}}$ viremia was cleared more quickly from the serum.

### Body weights

Body weights were collected weekly and used to interpolate daily weights. To obtain separate weight gain curves for each pig, a random regression model was fitted to the weight data of all animals, separately for the NVSL and KS06 trials, using second order Legendre polynomials in the following model that was implemented in ASReml 3.0 [28]:

$$\begin{aligned} {\text{W}}_{ijklmop} \left( {\text{t}} \right) & = \mathop \sum \limits_{{{\text{n}} = 0}}^{2} {\text{L}}_{\text{ni}} \left( {\text{t}} \right) + {\text{P}}_{j} + {\text{A}}_{k} + {\text{S}}_{l} \\ & \quad + \mathop \sum \limits_{{{\text{n}} = 0}}^{2} {\text{L}}_{\text{ni}} \left( {\text{t}} \right) * {\text{R}}_{m} + \mathop \sum \limits_{{{\text{n}} = 0}}^{2} {\text{L}}_{\text{ni}} \left( {\text{t}} \right) * {\text{An}}_{i} + {\text{Tr}}_{o} + {\text{Pen}}\left( {\text{Tr}} \right)_{po} + {\upvarepsilon }_{ijklmop} , \\ \end{aligned}$$

where ${\text{L}}_{\text{ni}} \left( {\text{t}} \right)$ denotes the nth order Legendre polynomial at t dpi for individual i. ${\text{L}}_{\text{n}} \left( {\text{t}} \right)$, P, A, S and ${\text{L}}_{\text{ni}} \left( {\text{t}} \right) * {\text{R}}_{ }$ were fitted as fixed effects. ${\text{L}}_{\text{ni}} \left( {\text{t}} \right)$ was fitted as a covariate, with t ranging from 0 to 42 dpi, P is the parity of dam, classified as first, second, or later parities, A is the age of the individual at inoculation, S is the sex of the individual, and ${\text{L}}_{\text{ni}} \left( {\text{t}} \right) * {\text{R}}_{ }$ is the interaction between the nth order Legendre polynomial at t dpi (${\text{L}}_{\text{ni}} \left( {\text{t}} \right)$) and rebound status (R). ${\text{L}}_{\text{ni}} \left( {\text{t}} \right) * {\text{An,}}$ Tr, and ${\text{Pen}}\left( {\text{Tr}} \right)$ were included as random effects and denote the interaction between the nth order Legendre polynomial at t dpi and animal, trial, and the nested effect of pen within trial, respectively. The term ${\text{L}}_{\text{n}} \left( {\text{t}} \right) * {\text{An}}_{ }$ models an individual’s weight at each time point and captures both genetic and permanent environmental effects, with an unstructured variance–covariance structure for polynomial parameters of a given animal and independence of parameters between animals. Residual variances were modeled separately for each dpi, in order to allow for an increase in variance over time. Trial and Trial * Pen were included to capture systematic environmental effects. This model was then used to obtain fitted values of each pig’s weight for each dpi (0 to 42) ($(\widehat{{{\text{W}}\left( {\text{t}} \right)}})$, using all coefficients estimated from the above model.

### Genomic relationship matrices

Due to the limited pedigree information and availability of genotypes on all animals with phenotypes, a genomic relationship matrix (**G**) was constructed from the 60k SNP genotype data, using the method of VanRaden [30]. The **G**-matrix included relationships among all animals across trials. In some cases, fitting relationships between breeds can absorb between-breed differences that could be due to selection, which can overestimate the genetic variance because the “base population” is the population from which the breeds subsequently diverged [31]. Thus, a block diagonal **G**-matrix was also constructed $\left( {{\mathbf{G}}_{{\mathbf{B}}} } \right)$ that only considered relationships between animals from the same genetic background, with zero relationships between animals from different companies. Results from analyses with ${\mathbf{G}}_{{\mathbf{B}}}$ are expected to be similar to what would be found with a pedigree-based analysis of these data if the pedigree was more extensive. A third **G**-matrix was constructed that was the same as ${\mathbf{G}}_{{\mathbf{B}}}$ but only included animals from trials that were paired across isolates (${\mathbf{G}}_{{\mathbf{P}}}$), to assess whether the estimates of correlations of traits between NVSL and KS06 infected pigs could be biased due to different breed crosses being evaluated for each isolate. In order to assess the impact of the WUR SNP on these genetic correlations, matrices **G**, ${\mathbf{G}}_{{\mathbf{B}}}$ and ${\mathbf{G}}_{{\mathbf{P}}}$ were also constructed after excluding the 118 SNPs in the 5 Mb region surrounding the WUR SNP. These new matrices were designated as ${\mathbf{G}}_{{ - {\mathbf{W}}}} ,\,{\mathbf{G}}_{{{\mathbf{B}} - {\mathbf{W}}}}$ and ${\mathbf{G}}_{{{\mathbf{P}} - {\mathbf{W}}}}$, respectively.

### Statistical models for phenotypic and genetic comparisons of NVSL and KS06

All analyses used to evaluate responses to NVSL and KS06 infections were conducted using an animal model in ASReml 3.0 [28]. The univariate model was:

$$\begin{aligned} {\text{Y}}_{ijklmnopq} & = {\upmu } + {\text{P}}_{i} + {\text{A}}_{j} + {\text{W}}_{k} + {\text{S}}_{l} \\ & \quad + {\text{R}}_{m} + {\text{An}}_{n} + {\text{Li}}_{o} + {\text{Tr}}_{p} + {\text{Pen}}\left( {\text{Tr}} \right)_{qp} + \varepsilon_{ijklmnopq} , \\ \end{aligned}$$

where Y is the dependent variable of daily fitted viremia values, fitted weights, VL, TP, PV, ${\text{Tmax,}}\,{\text{Vmax}}$, or weight gain from 0 to 42 dpi (WG). Parity of the dam (P), classified as first, second, or later parity, and sex of the piglet (S) were fitted as a fixed class effect. To account for potential model differences in curve fittings between rebound and non-rebound pigs, the fixed class effect of rebound (R) was included in the model. Age (A) and weight (W) of the piglet at infection (0 dpi) were included as linear covariates. Random effects included animal genetic effects (An; using the full **G**-matrix), litter (Li), trial (Tr), Pen nested within trial (${\text{Pen}}\left( {\text{Tr}} \right)$), and ${\upvarepsilon }$ as the residual. The random effect of animal, An, was assumed to have a variance–covariance structure proportional to the genomic relationship matrix generated from SNP genotypes. Normal distributions of the error and random effect of animal were assumed $\sim\;N\left( {0,{\mathbf{I}}{\upsigma }_{\upvarepsilon } } \right)$ and $\sim\;N\left( {0,{\mathbf{G}}{\upsigma }_{\text{g}} } \right),$ respectively. The phenotypic variance was obtained by summing the animal, litter, and pen within-trial variance components. Heritability was obtained by dividing the animal variance component by the phenotypic variance.

#### Comparison of host response to NVSL and KS06 infection

Data from paired trials (Table 1) and the full **G**-matrix were used to estimate the effect of isolate on daily fitted viremia values, weekly weights, VL, TP, PV, ${\text{Tmax,}}\,{\text{Vmax}}$ or WG by including isolate as a fixed class effect into the above model. Phenotypic differences between virus isolates were assessed using the t-statistic reported by ASReml 3.0 [28], with a significance cutoff of α = 0.05.

#### Genetic parameters by isolate

In order to quantify relationships between the response traits, heritabilities and phenotypic and genetic correlations were estimated separately for each isolate for VL, WG, TP, PV, ${\text{Tmax}}$, and ${\text{Vmax}}$, using the full **G**-matrix. Heritabilities and litter effects were estimated using a univariate model. A multivariate model using all traits was attempted for genetic and phenotypic correlations between traits but this model did not achieve convergence, so bivariate models were used instead. Estimates of correlations were considered statistically significant at α = 0.05 based on a t-test with 1496 degrees of freedom for NVSL and 670 degrees of freedom for KS06.

The genetic correlation between viremia and weight gain was expected to change during the course of infection. Thus, the genetic correlation between $\widehat{{{\text{V}}\left( {\text{t}} \right)}}$ and 3-day weight gain at day t ($\widehat{{{\text{WG}}\left( {\text{t}} \right)}}$) were estimated for every other dpi (i.e. 1, 3, 5, …, 41), separately for each isolate, using a bivariate model. Three-day weight gain was derived from the fitted daily weights and included weight 3 days before as a covariate instead of weight at day zero. Weight gain at 1 dpi was adjusted for weight at infection. These bivariate analyses resulted in two 21 × 21 matrices of genetic correlations between viremia and 3-day weight gain, which were visualized in heat maps.

#### Association of the WUR genotype with response to NVSL and KS06 infection

Associations of the genotype at the WUR SNP with VL, WG, TP, PV, ${\text{Tmax}}$, and ${\text{Vmax}}$, were estimated separately for infection with NVSL and KS06 by including the interaction of isolate with WUR genotype into the above model, with the full **G**-matrix representing the relationships between animals. This model was also fitted to daily fitted viremia values and fitted weights to generate viremia and weight curves for each isolate and WUR genotype. For these analyses, alleles at the WUR SNP were reported using the Illumina A/B genotype reference system, as was used in the original studies that reported the association of this SNP with host response following PRRSV infection [9–11]. Statistical differences between each isolate by WUR genotype combination were assessed using the t-statistic reported in ASReml [28] and the residual degrees of freedom from the model, with a significance cutoff of α = 0.05.

#### Genetic correlations of response between isolates

The different **G**-matrices described above were used to estimate genetic correlations of VL, WG, TP, PV, ${\text{Tmax}}$, and ${\text{Vmax}}$ between the two virus isolates using a bivariate model. Genetic correlations were evaluated for statistical significance based on a t-test with 2168 degrees of freedom when using **G**, ${\mathbf{G}}_{{ - {\mathbf{W}}}}$, ${\mathbf{G}}_{{\mathbf{B}}}$, and ${\mathbf{G}}_{{{\mathbf{B}} - {\mathbf{W}}}}$, and with 1378 degrees of freedom when using only paired trials (${\mathbf{G}}_{{\mathbf{P}}}$ and ${\mathbf{G}}_{{{\mathbf{P}} - {\mathbf{W}}}}$).

## Results

### Comparison of host response to infection with NVSL and KS06

**Fig. 1:**
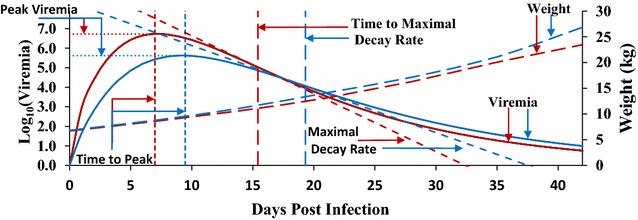
Comparison of response to infection when pigs are infected with NVSL (*red*) or KS06 (*blue*) PRRSV isolates. For comparison between viruses, trials were matched based on genetic background. Least square means of daily viremia, predicted using the monophasic Wood’s curve parameters, and weights were estimated using ASReml [28] with the full **G**-matrix. Viremia, Weight, Time to Peak, Peak Viremia, Time to Maximal Decay, and Maximal Decay were compared when pigs were infected with either the NVSL or KS06 PRRSV isolate

Raw viremia profiles suggested differences in pig response to infection with the NVSL versus the KS06 PRRSV isolate (see Additional file 1: Figure S1). To statistically quantify these differences, a selection of curve characteristics were derived from the Wood’s function parameters and compared between isolates using data from trials that were paired by genetic background to remove confounding between isolate and genetic background (Table 1). Pigs infected with NVSL had 16 ± 2 % higher VL than pigs infected with KS06 (Table 3; Fig. 1). Pigs infected with NVSL had 14 ± 2 % higher PV and reached PV 2.5 ± 0.6 days earlier (TP) than pigs infected with KS06 (Table 3; Fig. 1). Compared to pigs infected with KS06, NVSL-infected animals reached maximal PRRSV clearance 3.9 ± 0.7 days earlier (${\text{Tmax}}$) and cleared at a 36 ± 14 % faster maximal rate (${\text{Vmax}}$) than their KS06-infected counterparts (Table 3; Fig. 1). When comparing the impact that infection had on weight gain, pigs infected with the NVSL isolate had a tendency to grow slower than their KS06-infected counterparts (Table 3; Fig. 1). This comparison of viremia characteristics and WG between isolates (Table 3; Fig. 1) indicates that NVSL is more virulent than KS06 because it reached a higher PV more rapidly and resulted in higher VL and slower growth of the pigs. KS06 appears to be more persistent than NVSL, as shown by a longer time to maximal decay rate, lower maximal decay, and a larger percentage of pigs classified as persistently infected, defined as a non-rebound pig with a fitted log_10_ serum viremia value greater than 1 at 42 dpi (56 % for KS06 vs. 40 % for NVSL; Additional file 2: Table S1).

**Table 3:** Least square means, heritabilities, litter effects, and phenotypic standard deviations (SD) of responses to infection with the NVSL and KS06 isolates

Trait	LS means^a,b^		P value^c^	Heritability^b,d^		Litter^b,d^		Phenotypic sd^b,d^	
	NVSL (se)	KS06 (se)		NVSL (se)	KS06 (se)	NVSL (se)	KS06 (se)	NVSL	KS06
WG	15.8 (1.1)	19.5 (1.4)	0.076	0.33 (0.06)	0.31 (0.09)	0.07 (0.03)	0.03 (0.04)	3.90	3.91
VL	110.5 (1.4)	95.0 (1.6)	<0.001	0.31 (0.06)	0.51 (0.09)	0.24 (0.03)	0.01 (0.04)	7.90	7.46
TP	7.0 (0.4)	9.5 (0.4)	0.004	0.22 (0.05)	0.20 (0.09)	0.16 (0.03)	0.10 (0.05)	1.36	1.54
PV	6.6 (0.1)	5.8 (0.1)	<0.001	0.17 (0.05)	0.45 (0.08)	0.27 (0.04)	0.00 (0.00)	0.40	0.41
Tmax	15.4 (0.5)	19.3 (0.6)	0.002	0.21 (0.05)	0.16 (0.09)	0.15 (0.03)	0.14 (0.05)	2.38	2.73
Vmax	0.30 (0.02)	0.22 (0.02)	0.033	0.09 (0.05)	0.26 (0.09)	0.08 (0.03)	0.01 (0.04)	0.08	0.05

*WG* weight gain (kg), *VL* viral load (area under the Wood’s curve of log_10_ serum viremia from 0 to 21 days post-infection; viremia * days), *TP* time to peak viremia (days), *PV* peak viremia (log_10_ serum viremia), *Tmax* time to maximal rate of viremia decay (days), *Vmax* maximal rate of viremia decay (log_10_ serum viremia/day), *se* standard error

^a^Full **G**-matrix used

^b^Estimates were obtained by fitting isolate in the model and only included trials in which pigs from the same genetic background were infected with both NVSL and KS06

^c^P value for the difference in the estimated least square (LS) means between NVSL and KS06

^d^Estimates were obtained by using the full **G**-matrix; NVSL and KS06 estimates were estimated separately, and included all animals infected with that isolate, except trial 13

### Heritability estimates for viremia curve characteristics and weight gain

All evaluated traits were estimated to be moderately to highly heritable, except for ${\text{Vmax}}$ under infection with NVSL (Table 3). The traits with the highest estimated heritability under infection with NVSL were VL and WG and these traits also had high heritability estimates under infection with KS06. ${\text{Vmax}}$ had the lowest estimated heritability under infection with NVSL compared to the other PRRSV curve characteristics, but a moderate heritability under infection with KS06 (Table 3). The estimated genetic variance for ${\text{Vmax}}$ was similar under infection with NVSL and KS06 (0.00053 vs. 0.00072), so the difference in heritability was primarily driven by a larger environmental variance under infection with NVSL compared to KS06 (0.0057 vs. 0.0027).

Heritability estimates were similar between NVSL and KS06 for WG, TP, and ${\text{Tmax}}$ (Table 3). These traits also had similar estimates of the litter component for NVSL and KS06 infected pigs. Traits VL, PV, and ${\text{Vmax}}$ had lower heritabilities and larger litter components for NVSL compared to KS06. Summing the heritability and litter components gave similar results for the two isolate for VL and PV, although heritabilities were quite different between isolates (Table 3). The larger number of animals infected with the NVSL isolate compared to KS06 may result in more accurate separation of genetic and litter components for NVSL.

### Genetic parameter estimates using different G matrices

Estimates of the genetic correlation between isolates for PV were very similar when using the full **G** matrix (**G**) or the **G** matrix with only genetic relationships within genetic background (${\mathbf{G}}_{{\mathbf{B}}}$; Table 4), indicating that there is little variation in host genetic factors influencing PV between genetic backgrounds. The genetic correlation between isolates for WG was slightly higher when using ${\mathbf{G}}_{{\mathbf{B}}}$ compared to **G** (Table 4), suggesting that the impact of PRRSV infection on WG of pigs within the same genetic background was more similar than between pigs of different genetic backgrounds. The estimate of the genetic correlation of VL between pigs infected with NVSL and KS06 dropped substantially when ${\mathbf{G}}_{{\mathbf{B}}}$ was used, compared to using **G** (Table 4). The estimate of genetic correlation between isolates for TP increased when using ${\mathbf{G}}_{{\mathbf{B}}}$ rather than **G** (Table 4), such that the estimate based on ${\mathbf{G}}_{{\mathbf{B}}}$ was no longer significantly different from 1 but also not significantly different from 0. Estimates of the genetic correlation between isolates for ${\text{Tmax}}$ and ${\text{Vmax}}$ were not significantly different from 0 or 1 when using either ${\mathbf{G}}_{{\mathbf{B}}}$ or **G** (Table 4). In general, estimates obtained when including only relationships within genetic background were similar, whether all animals were used (${\mathbf{G}}_{{\mathbf{B}}}$) or only those from trials that were paired across isolates (${\mathbf{G}}_{{\mathbf{P}}}$).

**Table 4:** Estimates of genetic correlations [genet cor. (standard errors)] between response to infection with the NVSL versus KS06 isolates and using different relationship matrices

Trait	Full (**G**)			Block diagonal (**G**_**B**_)			Paired block diagonal (**G**_**p**_)		
	Heritability		Genet cor.	Heritability		Genet cor.	Heritability		Genet cor.
	NVSL	KS06		NVSL	KS06		NVSL	KS06	
VL	0.32 (0.06)	0.53 (0.07)	0.86 (0.19)	0.40 (0.06)	0.53 (0.08)	0.51 (0.24)	0.51 (0.08)	0.54 (0.09)	0.57 (0.22)
WG	0.33 (0.05)	0.30 (0.09)	0.86 (0.27)	0.37 (0.06)	0.32 (0.10)	0.96 (0.34)	0.41 (0.08)	0.38 (0.11)	0.90 (0.31)
TP	0.22 (0.05)	0.21 (0.09)	0.25 (0.33)	0.28 (0.06)	0.28 (0.10)	0.40 (0.36)	0.32 (0.08)	0.30 (0.12)	0.43 (0.36)
PV	0.17 (0.05)	0.46 (0.07)	0.94 (0.28)	0.23 (0.06)	0.43 (0.08)	0.94 (0.33)	0.29 (0.07)	0.42 (0.09)	0.91 (0.30)
Tmax	0.21 (0.05)	0.14 (0.09)	0.82 (0.53)	0.26 (0.06)	0.16 (0.10)	0.86 (0.59)	NE^a^	NE	NE
Vmax	0.10 (0.05)	0.25 (0.09)	0.63 (0.51)	0.13 (0.05)	0.22 (0.10)	0.32 (0.67)	0.06 (0.06)	0.23 (0.12)	0.41 (0.92)

**G** = full **G**-matrix with all relationships included; **G**_**B**_ = block diagonal **G**-matrix, with the relationships between animals from different genetic backgrounds set to zero; **G**_**p**_ = paired block diagonal **G**-matrix, with the relationships between animals from different genetic backgrounds set to zero, and only included trials in which pigs from the same genetic background were infected with both NVSL and KS06

*WG* weight gain (kg), *VL* viral load (area under the Wood’s curve of log_10_ serum viremia from 0 to 21 days post infection; viremia * days), *TP* time to peak viremia (days), *PV* peak viremia (log_10_ serum viremia), *Tmax* time to maximal rate of viremia decay (days), *Vmax* maximal rate of viremia decay (log_10_ serum viremia/day)

^a^NE: were not estimated because the model did not achieve convergence in ASReml

### Genetic correlations among viral load and viremia curve characteristics

VL, defined as area under the Wood’s curve from 0 to 21 dpi, was largely driven by PV, as shown by the high genetic and phenotypic correlations between these two traits for both isolates (Tables 5, 6). PV had the highest genetic correlation between PRRSV isolates and was not significantly different from 1 (Table 4). No other curve characteristic had a between virus isolate genetic correlation estimate that was significantly different from 0. This suggests that the observed genetic correlation between isolates for VL is primarily due to the high genetic correlation observed between isolates for PV.

**Table 5:** Estimates of correlations^a^ (standard error) of response to infection with PRRSV isolate NVSL

Trait	VL	WG	TP	PV	Tmax	Vmax
VL		−0.33 (0.03)	0.10 (0.03)	0.66 (0.02)	0.36 (0.03)	−0.27 (0.03)
WG	−0.74 (0.10)		−0.02 (0.03)	−0.22 (0.03)	−0.16 (0.03)	0.12 (0.03)
TP	0.31 (0.15)	0.27 (0.16)		−0.09 (0.03)	0.72 (0.01)	0.12 (0.03)
PV	0.85 (0.07)	−0.73 (0.13)	0.05 (0.19)		−0.23 (0.03)	0.40 (0.03)
Tmax	0.81 (0.10)	−0.11 (0.16)	0.83 (0.07)	0.50 (0.21)		−0.51 (0.02)
Vmax	−0.72 (0.21)	0.45 (0.22)	−0.11 (0.26)	−0.27 (0.33)	−0.57 (0.19)	

*WG* weight gain (kg), *VL* viral load (area under the Wood’s curve of log_10_ serum viremia from 0 to 21 days post infection; viremia * days), *TP* time to peak viremia (days), *PV* peak viremia (log_10_ serum viremia), *Tmax* time to maximal rate of viremia decay (days), *Vmax* maximal rate of viremia decay (log_10_ serum viremia/day)

^a^Phenotypic correlations (above diagonal) and genetic correlations (below diagonal) were estimated using an animal model in ASReml and the full **G**-matrix

**Table 6:** Correlations of response^a^ to infection with PRRSV isolate KS06 using the full **G**-matrix

Trait	VL	WG	TP	PV	Tmax	Vmax
VL		−0.23 (0.05)	−0.06 (0.05)	0.76 (0.02)	0.13 (0.05)	−0.16 (0.05)
WG	−0.52 (0.17)		−0.05 (0.05)	−0.13 (0.05)	−0.06 (0.06)	0.13 (0.05)
TP	−0.08 (0.22)	−0.10 (0.24)		0.02 (0.05)	0.80 (0.02)	0.02 (0.05)
PV	0.91 (0.05)	−0.30 (0.18)	−0.08 (0.25)		−0.19 (0.05)	0.52 (0.04)
Tmax	0.19 (0.23)	−0.42 (0.23)	0.69 (0.19)	−0.24 (0.28)		−0.52 (0.04)
Vmax	−0.01 (0.20)	0.42 (0.21)	−0.12 (0.28)	0.51 (0.13)	−0.75 (0.18)	

*WG* weight gain (kg), *VL* viral load (area under the Wood’s curve of log_10_ serum viremia from 0 to 21 days post infection; viremia * days), *TP* time to peak viremia (days), *PV* peak viremia (log_10_ serum viremia), *Tmax* time to maximal rate of viremia decay (days), *Vmax* maximal rate of viremia decay (log_10_ serum viremia/day)

^a^Phenotypic correlations (above diagonal) and genetic correlations (below diagonal) were estimated using an animal model in ASReml and the full **G**-matrix

Tmax and Vmax had strong negative genetic correlations with each other for both isolates but they were only highly correlated with VL for NVSL (Tables 5, 6). Time to maximal decay rate (${\text{Tmax}}$) was 19.3 days for KS06 but 15.4 days for NVSL (Table 3). Thus, ${\text{Vmax}}$ was expected to play a larger role in VL for NVSL than for KS06, because VL was calculated from 0 to 21 dpi. No conclusions can be drawn about the genetic correlations between isolate for ${\text{Tmax}}$ or ${\text{Vmax}}$ because the estimates were not significantly different from 0 or 1 due to large standard errors (Table 4).

The two time-related traits TP and ${\text{Tmax}}$ had strong positive genetic and phenotypic correlations with each other for both isolates (Tables 5, 6) because TP $\left( {\frac{\text{b}}{\text{c}}} \right)$ is a component of ${\text{Tmax}}\,\left( {{\text{Tmax}} = {\text{TP}} + \frac{{\sqrt {\text{TP}} }}{{\sqrt {\text{c}} }}} \right)$. The genetic correlation between isolates was significantly different from 1 for TP (0.25 ± 0.33, Table 4), indicating that host genetic control of the time until maximal virus decay rate may differ between virus isolates.

### Genetic correlations of weight gain with viremia curve characteristics

PV had a moderate negative genetic correlation with WG for NVSL-infected pigs (Table 5) but this genetic correlation was not significantly different from 0 for KS06-infected pigs (Table 6), due to a larger standard error and a less negative estimate. ${\text{Vmax}}$ also had a significant genetic correlation with WG. These results suggest that the reduction in growth is caused by an overall high viremia level over a prolonged period of time, which is further supported by the finding that WG had the highest estimated genetic and phenotypic correlations with VL for both isolates (Tables 5, 6).

### Genetic correlations of viremia with weight gain

The genetic and phenotypic correlations between VL and WG were negative and of similar magnitude for the two isolates (Tables 5, 6). Between isolates, genetic correlation estimates for VL and WG were high and not significantly different from 1 (Table 4), indicating that host genetic control of VL and WG was very similar under infection with either the NVSL or KS06 isolate.

**Fig. 2:**
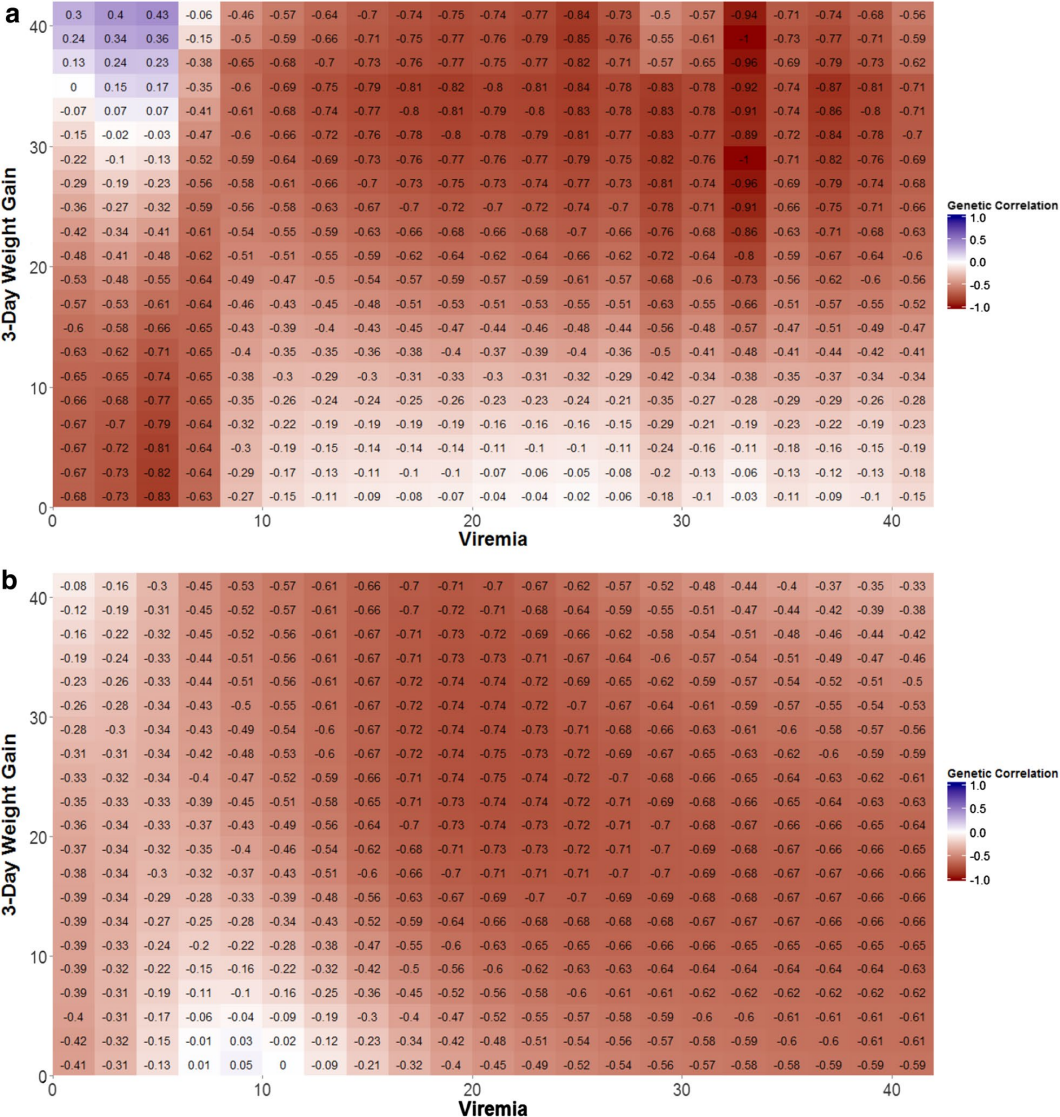
Heat Map of genetic correlations between viremia and weight gain during the course of infection with the **a** NVSL or **b** KS06 PRRSV isolate. Genetic correlations from fitting a bivariate animal model in ASReml [28] using the full **G**-matrix. NVSL and KS06 were analyzed separately. All trials, except trial 13, were used in the analysis. Each *square* in the heat map represents the genetic correlation between viremia at a given time point t (*X* axis) and the 3-day weight gain at time point t* (*Y* axis)

A more thorough exploration of the relationship between PRRS viremia and weight gain was obtained by estimating genetic correlations between fitted viremia and 3-day weight gain across the infection period (Fig. 2). Genetic correlations generally showed a similar pattern between NVSL and KS06, but correlations for NVSL were more extreme (range = −1 to 0.43) than KS06 (range = −0.75 to 0). A more detailed explanation of these results can be found in the Discussion section ‘Genetic correlation of viremia with weight gain across time’.

### Associations of the WUR genotype with response to infection with NVSL and KS06

**Fig. 3:**
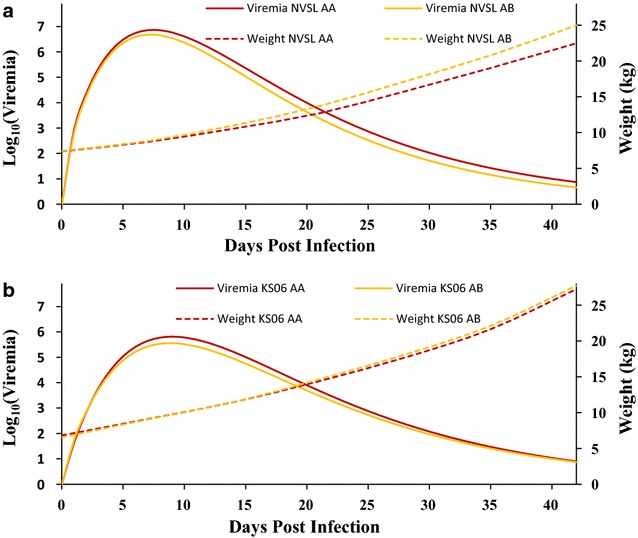
Least square means of the effect of the WUR genotype for predicted viremia and weight in pigs infected with either the NVSL or KS06 PRRSV isolate. Least square means of the WUR genotype for predicted viremia and weight for NVSL (**a**) and KS06 (**b**) when fitting the Isolate * WUR interaction into the animal model in ASReml [28] using the full **G**-matrix. All trials, except trial 13, were used for the analysis

Least square means of daily viremia and weight were estimated by fitting the interaction between isolate and WUR genotype for all trials simultaneously and are in Fig. 3. Very few pigs had the BB genotype at the WUR SNP, so estimates of least square means for the BB genotype had high standard errors. For VL, both AB and BB animals were significantly different from AA, while BB animals were not significantly different from AB, suggesting complete dominance, as previously reported by Boddicker et al. [9–11]. Results for BB animals are not discussed further.

**Fig. 4:**
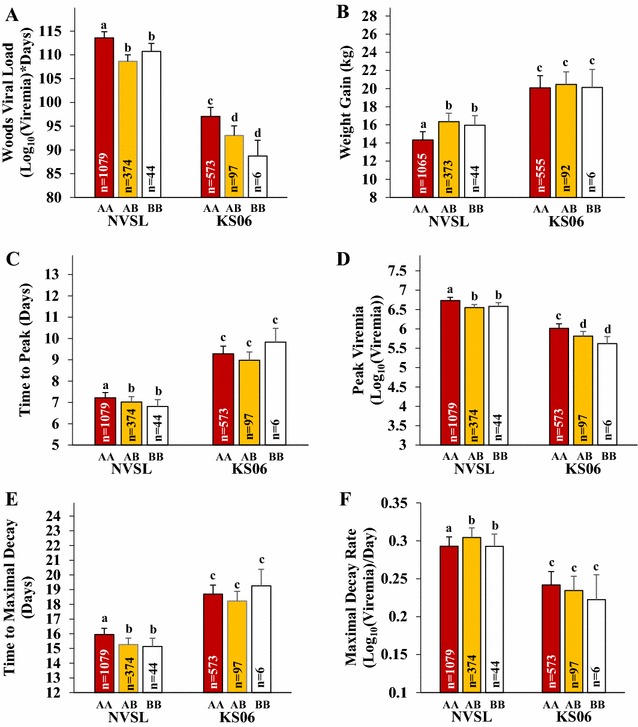
Least square means of the effect of the WUR genotype for weight gain and viremia curve parameters in pigs infected with either the NVSL or KS06 PRRSV isolate. Least square means of the WUR genotype for VL (**A**), WG42 (**B**), TP (**C**), PV (**D**), Tmax (**E**), and Vmax (**F**) when fitting the Isolate * WUR interaction into the animal model in ASReml [28] using the full **G**-matrix. All trials, except trial 13, were used for the analysis. Estimates with *different letter* assignments are significantly different (P ≤ 0.05)

Pigs with the AA WUR genotype had 4.5 ± 0.4 % higher VL (P < 0.001; Fig. 4A) and grew 2.0 ± 0.2 kg less than pigs with the AB genotype after infection with NVSL (P < 0.001; Fig. 4B). These estimates are consistent with previous estimates of the association of WUR under infection with NVSL [11]. Genotype at the WUR SNP was also found to be associated with VL under infection with KS06, for which VL was 4.2 ± 0.9 % higher in AA animals than in AB animals (P < 0.001) (Fig. 4A). However, in contrast to infection with NVSL, the WUR genotype did not have a significant association with WG (P = 0.32), although the direction of the effect was consistent, with AA pigs growing 0.4 ± 0.4 kg less than pigs with the AB genotype (Fig. 4B).

Genotype at the WUR SNP was associated with all viremia curve characteristics in pigs infected with NVSL (Figs. 3, 4A). Compared to AA animals, AB animals had 2.8 ± 0.4 % lower PV (P < 0.001; Fig. 4D), which was reached 0.20 ± 0.09 days earlier (P < 0.02; Fig. 4C). AB animals also had a 3.8 ± 1.5 % faster maximal decay rate (P < 0.02; Fig. 4F), which was reached 0.68 ± 0.16 days sooner (P < 0.001; Fig. 4E). In KS06 trials, genotype at the WUR SNP was associated with 3.4 ± 0.7 % higher PV in AA animals compared to AB animals (P < 0.001; Fig. 4D) but no association was found with ${\text{Vmax}}$ (P = 0.36; Fig. 4F) and the direction of the effect for ${\text{Vmax}}$ was opposite to that of NVSL-infected animals, with AB having a 3.1 ± 3.4 % slower maximal decay rate than AA animals. Compared to AA animals, AB animals tended to reach peak viremia 0.30 ± 0.16 (P = 0.052) days sooner (Fig. 4C) and the maximal decay rate 0.47 ± 0.29 (P = 0.078) days later (Fig. 4E). The effect of WUR genotype was significantly different between the NVSL and KS06 isolates for either WG (P = 0.001) and ${\text{Vmax}}$ (P = 0.041; Fig. 4).

Plotting average weight curves and viremia using the Wood’s curve parameters from the primary phase of infection (i.e. not including rebound) for pigs with the AA and AB genotypes at the WUR SNP by isolate (Fig. 3), provides a visualization of the overall differences in the shape of the viremia and weight curves. For KS06, the effect of the WUR genotype on VL was mainly driven by differences in PV but the difference in viremia level between AA and AB was not maintained due to a slightly lower rate of clearance in AB compared to AA animals, resulting in similar viremia levels at 42 dpi. Conversely, for NVSL, the difference in viremia levels between AA and AB animals first appeared around peak viremia and became larger during the primary stages of infection due to a faster clearance rate for AB animals. In general, WUR genotype had a lower association with response to infection in KS06-infected pigs than in NVSL-infected pigs, which suggests that the magnitude of the effect of QTL on chromosome 4 depends on virulence of the PRRSV isolate.

### Impact of the WUR region on heritabilities and genetic correlations

Heritabilities of response traits were estimated by including all SNPs in the full **G**-matrix (Table 4) and also by excluding SNPs in the 5 Mb region surrounding the WUR SNP (${\mathbf{G}}_{{ - {\mathbf{W}}}}$; Table 7) in order to assess how much of the estimates of heritability were attributed to the WUR genotype. In the NVSL trials, estimated heritabilities were lower for all traits when the ${\mathbf{G}}_{{ - {\mathbf{W}}}}$ was used, except for ${\text{Vmax}}$, which remained the same. Heritability estimates for WG and ${\text{Tmax}}$ were not affected by using ${\mathbf{G}}_{{ - {\mathbf{W}}}}$, while the estimates dropped for all other traits when ${\mathbf{G}}_{{ - {\mathbf{W}}}}$ was used, with the largest drops for PV and VL.

**Table 7:** Estimates of genetic correlations of response to infection between PRRSV isolates when excluding the 5 Mb WUR region from the G matrix

Trait	Full (**G**_**−W**_)			Block diagonal (**G**_**B–W**_)			Paired block diagonal (**G**_**P–W**_)		
	Heritability		Genet cor.	Heritability		Genet cor.	Heritability		Genet cor.
	NVSL	KS06		NVSL	KS06		NVSL	KS06	
VL	0.25 (0.06)	0.49 (0.09)	0.76 (0.22)	0.34 (0.06)	0.49 (0.08)	0.44 (0.26)	0.45 (0.08)	0.49 (0.09)	0.51 (0.24)
WG	0.28 (0.06)	0.30 (0.09)	0.89 (0.29)	0.33 (0.06)	0.31 (0.07)	0.93 (0.35)	0.36 (0.08)	0.38 (0.11)	0.90 (0.32)
TP	0.21 (0.05)	0.20 (0.09)	0.18 (0.34)	0.27 (0.06)	0.28 (0.10)	0.36 (0.37)	0.31 (0.08)	0.29 (0.12)	0.37 (0.37)
PV	0.13 (0.05)	0.40 (0.08)	0.79 (0.34)	0.22 (0.06)	0.39 (0.08)	0.81 (0.37)	0.51 (0.10)	0.55 (0.36)	0.77 (0.36)
Tmax	0.19 (0.05)	0.14 (0.09)	0.80 (0.54)	0.24 (0.06)	0.16 (0.10)	0.90 (0.62)	NE^a^	NE	NE
Vmax	0.10 (0.05)	0.23 (0.09)	0.70 (0.53)	0.13 (0.05)	0.21 (0.09)	0.57 (0.70)	0.06 (0.07)	0.22 (0.11)	0.75 (1.03)

*WG* weight gain (kg), *VL* viral load (area under the Wood’s curve of log_10_ serum viremia from 0 to 21 days post infection; viremia * days), *TP* time to peak viremia (days), *PV* peak viremia (log_10_ serum viremia), *Tmax* time to maximal rate of viremia decay (days), *Vmax* maximal rate of viremia decay (log_10_ serum viremia/day)

**G**_**−W**_ = full **G**-matrix constructed excluding the 5 Mb region containing WUR with all relationships included; **G**_**B−W**_ = block diagonal **G**-matrix constructed excluding the 5 Mb region containing WUR, with the relationships between animals from different genetic backgrounds set to zero; **G**_**P−W**_ = paired block diagonal **G**-matrix constructed excluding the 5 Mb region containing WUR, with the relationships between animals from different genetic backgrounds set to zero, and only included trials in which pigs from the same genetic background were infected with both NVSL and KS06

^a^NE: were not estimated because the model did not achieve convergence in ASReml

Genetic correlations for the response traits between isolates were also estimated using **G** (Table 6) and ${\mathbf{G}}_{{ - {\mathbf{W}}}}$ (Table 7). Estimates of genetic correlations between isolates for WG and ${\text{Vmax}}$ were slightly larger when using ${\mathbf{G}}_{{ - {\mathbf{W}}}}$ compared to **G**, while the estimates for all other response traits decreased, with the largest decreases for PV and VL. Genetic correlation estimates between isolates were significantly different from 0 and not significantly different from 1 for VL, WG and PV when using either **G** or ${\mathbf{G}}_{{ - {\mathbf{W}}}}$ (Tables 4, 7). The high genetic correlation estimates for these traits when using ${\mathbf{G}}_{{ - {\mathbf{W}}}}$ indicates that the conserved host genetic response between isolates for VL and PV did not solely depend on the WUR genotype but has a large polygenic component. The increase in the genetic correlation between isolates for WG when using ${\mathbf{G}}_{{ - {\mathbf{W}}}}$ compared to **G** is consistent with the observed effect of the WUR genotype in pigs infected with NVSL and the much smaller effect in pigs infected with KS06. Estimates of genetic correlations between isolates for ${\text{Tmax}}$ and ${\text{Vmax}}$ had large standard errors when using either **G** or ${\mathbf{G}}_{{ - {\mathbf{W}}}}$, so no conclusions could be drawn because estimates were not significantly different from 0 or 1. Estimates of the genetic correlations between isolates for TP were significantly different from 1 but not significantly different from 0 for either **G** or ${\mathbf{G}}_{{ - {\mathbf{W}}}}$, indicating that the host’s genetic control of TP is not highly conserved across isolates.

Matrices ${\mathbf{G}}_{{\mathbf{B}}}$ and ${\mathbf{G}}_{{\mathbf{P}}}$ were also constructed without the 5-Mb WUR region (${\mathbf{G}}_{{{\mathbf{B}} - {\mathbf{W}}}}$ and ${\mathbf{G}}_{{{\mathbf{P}} - {\mathbf{W}}}}$, respectively). The results obtained using these matrices were as expected based on the differences between **G** and ${\mathbf{G}}_{{ - {\mathbf{W}}}}$ and were also consistent with the differences in the estimates when comparing the use of ${\mathbf{G}}_{{\mathbf{B}}}$ and ${\mathbf{G}}_{{\mathbf{P}}}$ to the use of **G** (Tables 4, 7).

## Discussion

Our results suggest that the KS06 PRRSV isolate is less virulent than NVSL but, importantly, that genetic selection for pigs with improved weight gain and reduced viral load under either PRRSV infection is expected to be effective across these PRRSV isolates. This study also affirmed the important influence of the WUR10000125 genomic region on pig chromosome 4 on host response to PRRSV. The effect of this genomic region was consistent between isolates for traits related to viremia. While AB animals gained slightly more weight than AA animals during infection with KS06, a significant difference between WUR genotypes was only observed for weight gain with NVSL, suggesting that the influence of this genomic region on weight gain may depend on virulence of the PRRSV isolate.

Consistent with the previously conducted studies of the PHGC, the animals used in this study were inoculated with PRRSV both intramuscularly and intranasally. The infection protocol is a standard challenge protocol designed to give every pig a consistent amount of virus. It also simulates the most likely routes for infection through needle sticks and intranasal exposure. Previous studies comparing routes of exposure have shown that the dosage used to inoculate pigs impacts the level of viremia in the pigs independently of the route of exposure, resulting in similar levels of antibody production [32]. Similarly, growth and antibody responses were similar between intranasally and simultaneously intranasally/intramuscularly vaccinated pigs [33]. Given that no differences in host response are expected between these methods, the approach used in this study was the most appropriate to ensure consistent levels of infection between pigs to provide more power to distinguish between animals that differ in their genetic merit for response to PRRSV infection.

### Modelling viremia using the Wood’s curve

This study demonstrates the utility of mathematical functions to assess the impact of host genetics and virus isolate on PRRS viremia kinetics. The Wood’s curve uses three parameters, a, b, and c, which are related to the overall level of viremia (a) and describe the shape of the curve (b, which is dominant pre-peak, and c, which is dominant post-peak) [27]. While other mathematical functions may more adequately model PRRS viremia during infection, the number of data points collected during these trials limited the use of more complex models. The Wood’s curve is a more useful method for comparing viremia kinetics than the LOESS smoothed fit used in previous analyses of these data [9–11]. The LOESS smoothed fit uses a parameter that indicates the degree of polynomial to fit to the data and a smoothing parameter for curve fitting [34], with the primary intent of filtering out noise from the data. The limitation of the LOESS fit, however, is that it does not lend itself to extracting fitted parameters that specify particular biological properties of a system that have important implications in understanding the dynamics of PRRSV infection. Although both methods adequately fitted the data, Wood’s curve parameters describe both the magnitude and shape of the curve, which can be used to explore different characteristics of the viremia curves. Exploring Wood’s curve characteristics can provide insight into important biological questions, such as which aspects of host response are under strongest genetic control and how selection for one curve characteristic may affect others, and thus the entire profile. The Wood’s function can also be used to explore the relationship between curve characteristics and other phenotypes, such as growth under infection. Furthermore, comparison of the extended Wood’s and Wood’s curve functions allowed for an objective method to separate primary infection from rebound infection viremia curves [14].

While the advantages of fitting a Wood’s curve to model the dynamics of PRRS viremia are clear, care must be taken in the interpretation of correlations between curve characteristics because strong correlations between these curve characteristics are in part an artifact of the Wood’s function and partly reflect true correlations between curve characteristics that are independent of the Wood’s function. For example, ${\text{Tmax}}$ and ${\text{Vmax}}$ are expected to have a high genetic correlation because both rely heavily on the b parameter of the Wood’s function.

### Genetic parameter estimates using different **G** matrices

Three different **G** matrices were constructed for both the full **G** matrix and the ${\mathbf{G}}_{{ - {\mathbf{W}}}}$ matrix. ${\mathbf{G}}_{{\mathbf{B}}}$ only contained relationships between animals from the same genetic background, with zeros for relationships between animals from different companies. Thus, while **G** contains information about genetic variance that exists within genetic background as well as between genetic backgrounds, ${\mathbf{G}}_{{\mathbf{B}}}$ only contains information about genetic variance within genetic background and is, therefore, more similar to the pedigree-based relationship matrix because there was no pedigree information between animals from different genetic backgrounds. ${\mathbf{G}}_{{\mathbf{P}}}$ was a block diagonal matrix that used only pigs from the same genetic background that were paired across isolates and was used to avoid biases in estimates that could result from including different breeds in the analyses for each isolate. In general, estimates using ${\mathbf{G}}_{{\mathbf{P}}}$, which considered only animals from trials that were paired across isolates, were consistent with estimates using ${\mathbf{G}}_{{\mathbf{B}}}$. This suggests that, while the genetic correlation between isolates for VL may be moderate within genetic backgrounds, some genetic backgrounds have high VL under infection with both NVSL and KS06, while some genetic backgrounds have low VL under infection with both NVSL and KS06, such that when the relationships between genetic backgrounds are considered (using **G**), the genetic correlation between isolates for VL increases. Selecting for improved VL during infection with one PRRSV isolate is likely to improve VL during infection with another PRRSV isolate, but the extent to which such selection is successful across isolates will likely differ between genetic backgrounds.

### Comparison of genetic parameter estimates for viral load and weight gain to previous estimates

Estimates of heritability of VL and WG during NVSL infection were slightly different from previously reported estimates using these data [11] (VL: 0.31 ± 0.06 vs. 0.44 ± 0.13; WG: 0.33 ± 0.06 vs. 0.29 ± 0.11) (Table 3). Differences between estimates can be attributed to the use of genomic rather than pedigree-based relationships, the inclusion of trial 15, and the addition of age and weight at infection as covariates in the model used in this study. Age and weight at infection are important to include in the analysis because pigs that are older or heavier at infection tend to be able to mount a stronger immune response [35, 36]. The use of genomic instead of pedigree-based relationships halved the standard errors of estimates because the **G** matrix captures relationships between animals more accurately, especially with limited pedigree information available.

For the NVSL-infected pigs, the genetic correlation estimate was more negative than previously reported (${\text{r}}_{\text{g}}$: −0.74 ± 0.10 vs. −0.46 ± 0.20; ${\text{r}}_{\text{p}}$: −0.33 ± 0.03 vs. −0.29 ± 0.03) [11], which can be attributed to the combination of using genomic versus pedigree relationships, the addition of trial 15, and the inclusion of age and weight at infection as covariates. Strong genetic correlations between VL and WG suggest that there are common genes or pathways that affect both of these traits, likely through more resistant pigs having less viremia and therefore less infection-induced reduction in WG.

### Impact of PRRSV genetic diversity on host response to PRRSV infection

#### Differences in viremia and weight gain during infection with NVSL versus KS06

Our study demonstrates that, in addition to being genetically distinct [16], NVSL and KS06 PRRSV isolates differ in both their virulence and resulting viremia profile characteristics. Infection with NVSL was characterized by reaching high peak viremia early, followed by a quick clearance of the virus, whereas with KS06 virions accumulated more slowly towards a lower peak viremia and took longer to clear from serum. Pig growth tended to be less stunted when pigs were infected with the KS06 isolate compared to the NVSL isolate (Table 3). This may be because piglets infected with the KS06 isolate do not need to put as much energy into eliminating the virus, thus allowing them to place more emphasis on growth. These results are consistent with resource allocation theory, which hypothesizes that trade-offs between competing traits (e.g. health and growth) are a consequence of limited resources (i.e. energy availability) [37]. Genetic correlations between viremia and weight gain changed during the course of infection and tended to be more extreme in NVSL-infected pigs than in KS06-infected pigs. This suggests that more energy is required to fight infection with NVSL (Fig. 2), which is supported by the lower weight gain observed in NVSL-infected pigs (Fig. 1). These findings are consistent with those of Doeschl-Wilson et al. [38], who showed that a temporary reduction in growth, due to greater investment in immune response during early infection, benefits growth in the long-term due to a shorter or less severe infection, or both. The resources that the animal diverts to immune response and the impact on weight gain depend on the pathogen load that the animal experiences and thus on virulence of the isolate [38].

#### Genetic correlations of viremia with weight gain across time

In animals infected with NVSL, animals that had high viremia from 0 to 7 dpi tended to have low WG early on but high WG later on in the trial. During the early stages of infection, pigs with high viremia may need to allocate more energy to fight the infection and away from growth, resulting in a negative genetic correlation between early viremia and early weight gain. Thus, animals with higher early viremia divert more resources to fighting infection early on, which pays off in the long run with higher weight gain at the end of the trial. This notion is supported by the weaker genetic correlations between early viremia and weight gain under infection with the less virulent isolate, KS06 (Fig. 2b), which resulted in lower pre-peak viremia (Fig. 1). It is also likely that pigs with high early viremia suffer a greater loss in appetite [39], which may further reduce early weight gain in these animals. The positive genetic correlation between early viremia and late weight gain could reflect a return to homeostasis after infection in these pigs [40]. These observations are consistent with findings from a modeling approach that systematically investigated the short- versus long-term effects of infection and genetic resistance on growth, and the role of nutrient allocation on the relationship between growth and pathogen load [38].

While the relationship between weight gain and early viremia may differ between isolates, the ability of the animal to effectively clear the virus from the serum is crucial for maintaining growth. This is evident from a block of highly negative genetic correlation estimates of viremia at 15 to 28 dpi with weight gain from 22 dpi onward in pigs infected with NVSL (Fig. 2a). The time period from 15 to 28 dpi corresponds to the time when pigs are clearing PRRSV from the serum the most rapidly. Strong genetic correlations of viremia after 28 dpi with later weight gain were identified, in particular for viremia at 33 dpi. This negative genetic correlation is likely the result of rebound pigs, since this time point corresponds to the average time that rebound pigs reach secondary peak viremia.

Similar to NVSL, KS06-infected pigs showed strong negative genetic correlations of viremia with weight gain at approximately the time of maximal viral clearance (Fig. 2). In KS06-infected pigs, this critical period seemed to be viremia after 17 to 28 dpi and weight gain after 17 dpi, which corresponds to the time period in which antibodies, specifically IgG, are produced at the highest rates. This suggests that the ability to clear the virus effectively may depend on the pig’s ability to mount a successful adaptive immune response [41].

#### Viral rebound

Rebound (i.e. a bi-modal viremia profile) was observed more frequently when pigs were infected with the NVSL isolate than with the KS06 isolate. One possible explanation for the observed rebound in viremia is the presence of quasi-species of the virus within the host. PRRSV has a very high mutation rate, estimated to be between 4.7 and 9.8 × 10^−2^ nucleotides/year, which is the highest rate reported for an RNA virus [42]. This high mutation rate causes within-host variation in the PRRSV genome [43], with each population of common variants termed a quasi-species. Pigs infected with NVSL had higher viremia than pigs infected with KS06 throughout most of the pre-rebound phase (Fig. 1). This greater level of viremia means that more replication and, thus, more mutations have occurred for NVSL, so there is likely a greater number of quasi-species present in animals infected with NVSL than with KS06. The greater the number of quasi-species, the greater the chance that a variant is able to escape host immune response and cause viral rebound [44]. Animals in the same pen can also transfer quasi-species between each other, whereby a quasi-species from one pig could be transferred to another pig and cause re-infection and viral rebound [43].

Alternatively, NVSL may avoid host immune response more effectively than KS06, possibly escaping humoral immune response by localizing to certain tissues. Previous research has identified that the tonsils are a primary source of PRRSV persistence [45, 46]. This may be due to an abundance of memory B-cells in the tonsil but absence of effector, plasma-producing, B-cells [47]. An abundance of PRRSV in tonsils may result in cyclical reappearance of circulating virus. If the ability of the virus to localize to tissue to escape immune response differs between isolates, this will be reflected in the tonsil viremia levels. Studies are underway to address this possibility.

#### Impact of the QTL on pig chromosome 4 on PRRS disease resistance

Consistent with previous reports, the WUR SNP was significantly associated with VL during PRRSV infection, in that animals with the AB genotype had lower VL than animals with the AA genotype [9–11]. The effect of the WUR genotype on VL appeared to be primarily driven by the pig’s ability to control the rate of virus replication, based on the large effect of the WUR genotype on PV. This was the only curve characteristic for which the WUR genotype had a significant effect in both NVSL- and KS06-infected pigs (Fig. 4), which is likely due to the role that the putative causative gene, *GBP5*, plays in the host’s immune response. *GBP5* plays a role in the innate immune response during infection, and animals that have the AA genotype appear to produce no functional GBP5 [12]. Specifically, GBP5 interacts with NLRP3 (NOD-like receptor family, pyrin domain containing 3) and undergoes tetramerization to promote inflammasome assembly [13].

Although the QTL on pig chromosome 4 appears to play a significant role in host response to PRRSV infection, there is a substantial polygenic component beyond this region for VL and PV. The WUR genotype explained 13 % of the genetic variance for VL [9–11]. Consistent with this finding, accounting for the WUR genotype did not remove all of the heritability of VL and, although the genetic correlation between isolates dropped when accounting for the WUR genotype, it remained high and was not significantly less than 1 (Table 7).

Interestingly, the WUR genotype was not found to be significantly associated with WG in pigs infected with KS06, although the effect was in the same direction as for NVSL, only smaller. The difference in VL between isolates was greater than the difference in VL between AA and AB genotypes, thus AA individuals infected with KS06 had lower VL than AB individuals infected with NVSL. This may mean that less energy is needed to fight the virus during infection with the KS06 isolate and, as a result, weight gain was less affected, as evidenced by the higher weight gain when pigs were infected with KS06 compared to NVSL. Thus, the effect of the WUR genotype on weight gain may only be substantial during infection with more virulent isolates of PRRSV due to the increased severity of infection. Isolate-specific QTL for resistance have been identified in a number of infection (fungal, bacterial, and viral) studies in plants [48–50] and in a study on Dengue virus in mosquitoes [51].

It is likely that the QTL on pig chromosome 4 affects the severity of infection and its effect on WG is through the increased resources that have to be allocated to fighting the infection when viremia is higher. The relationship between VL and reduction in WG may be non-linear, which may explain why the direction of the effect of the WUR genotype on WG was the same for both PRRSV isolates, but the magnitude of the effect differed. Given the number of genetic factors that can influence WG, it is likely that what is in common for WG between these two isolates are the polygenic effects, which would explain the high genetic correlation between isolates observed for WG although a significant effect of the WUR genotype on WG was not found in the KS06 trials (Table 7). The WUR region explained 9 % of the genetic variance for WG in the NVSL trials, while few other genomic regions explained more than 1 % of the genetic variance [10].

### Potential avenues of selection for increased resistance to PRRS

#### Selection on WUR genotype

This study shows that genotype at the WUR SNP is associated with VL and PV (Fig. 4A, D) across two distinct PRRSV isolates, which indicates that selection to increase the frequency of the B allele, corresponding to increased PRRS resistance, is expected to reduce PRRS VL across isolates. Reducing viral burden has the potential to decrease the costs associated with PRRS by reducing PRRS incidence because a lower viral burden may also reduce virus shedding, which reduces the chance that other pigs will be infected. Although the WUR genotype did not have a significant effect on WG under infection with KS06, the direction of the effect was the same as for the NVSL isolate, thus selection to increase the frequency of the B allele at the WUR SNP is expected to improve WG under infection with more virulent isolates of the virus, with no negative effect on WG with less virulent isolates (Fig. 4B). Given the suspected dominant nature of this QTL [9–11] and the associated putative quantitative trait nucleotide (QTN) [12], increasing the frequency of the B allele is expected to improve PRRSV resistance by increasing the frequency of AB and BB animals.

Selecting for the AB genotype at the WUR SNP has the potential to reduce VL and PV across breeds and isolates, however the amount of response is limited because the region on pig chromosome 4 explained only a portion of the genetic variance in host response. The response traits VL and PV, however, also have large polygenic effects that appear to be conserved across isolates and breeds and to be independent of the WUR genotype (Tables 4, 7). Thus, genomic selection for VL or PV, in combination with marker-assisted selection on the WUR genotype, may hold the greatest potential for improved resistance to PRRS.

#### Potential for genomic selection

Genomic selection uses markers spaced throughout the genome to predict the genetic merit of an individual. All host response traits investigated here had a moderate to high heritable genetic component (Table 3), suggesting that genomic selection for different aspects of host response to PRRSV infection is feasible. There were high genetic correlations between VL, WG and PV for both isolates, except for PV and WG for KS06 (Tables 5, 6), which suggests that genomic selection for one trait is likely to improve response for the other two. Genetic correlations of host response between isolates were also high for VL, WG and PV (Table 4), which indicates that genomic selection for response to one isolate will result in improvement across isolates.

A limitation of genomic selection is the size of the data that must be generated on a continual basis in order to ensure accurate prediction of breeding values. It has also been shown that prediction accuracy decreases as the number of generations between the training and prediction sets increases [52], so periodic re-training on new phenotypes and genotypes will be necessary.

#### Potential for selection on response to vaccination

While genomic selection for host response to PRRSV appears appealing, quality infection data on many animals are needed to obtain accurate predictions. In principle, information on naturally-infected commercial pigs can be fed back into the nucleus in order to make selection decisions based on host response in commercial pigs. However, several factors cannot be controlled in a natural infection setting, such as virus dosage, time since infection, and the age and weight of the pig at infection, which all have an effect on how the pig responds to infection.

Response to vaccination may be an attractive alternative method for providing phenotypes on host response to PRRS because all of the above factors can be controlled with vaccination. A major current PRRS vaccine is a modified live virus, which has reduced virulence compared to commonly occurring wild type isolates. Thus, vaccinated pigs have circulating viremia that can be measured, similar to infection with any natural PRRSV isolate [53]. Measuring response to vaccination overcomes the limitations of natural infections because it is a controlled infection. However, before this can be implemented in industry, it is necessary to evaluate the genetic correlation between response to vaccination and response to natural infection with a variety of isolates.

Response to vaccination could be evaluated by using viremia measured on a single serum sample at the average time for pigs to reach peak viremia after vaccination, which may serve as an approximation for peak viremia of the individual. This has the advantage of needing only a single serum sample, rather than multiple samples throughout infection, as is needed for VL. Our results show that PV is highly genetically correlated with VL under infection with both NVSL and KS06 (Tables 5, 6) and that genetic control of PV is expected to be highly conserved between isolates, both within breeds and across the North American pig population (Table 4). To implement this, the expected time to peak after vaccination must be assessed, along with the genetic correlation of viremia at this time point with VL after vaccination.

#### Further considerations

When considering selection for increased disease resistance, particularly one specific disease, the consequences of this selection must be assessed. A common question raised is whether the parasite will evolve so as to overcome the genetic changes in the host (i.e. parasite evolution). This is especially important with a rapidly mutating virus such as PRRSV. Simulation studies have shown that selection for quantitative resistance (i.e. a continuous scale of levels of resistance) will result in selection of more virulent forms of the parasite [54]. Likewise, vaccines that have high efficiency, but less than 100 %, will also select for more virulent forms of the parasite, as was observed with Marek’s disease in chickens [55]. Selection on a single disease resistance locus can be considered in this same context if the resistance allele does not sufficiently reduce the spread of the parasite. This consequence can be alleviated by genomic selection, as the combination of genetic factors that confer higher genetic resistance in one animal is less likely to be identical to that in another animal. This can be further alleviated by using multiple approaches to combat the disease. On another, although not unrelated, note, selection for general health, or generalized immune response could be used [56, 57], which may be beneficial for infections with different pathogens, while presumably preventing the immune response to be skewed towards specific types of pathogens, which could increase the vulnerability to others.

Another consideration is the impact that selection for increased resistance may have on production. Recent studies have discussed the impacts of the WUR genotype on production traits. Abella et al. [58] reported that AA animals have superior growth performance compared to AB animals in an uninfected setting, while another study [59] found that AB animals had equivalent or superior growth performance and meat quality compared to AA animals. However, further investigation on the broader impact of selection for improved response to PRRSV infection is needed.

There are two major types of PRRSV: type 1 (European) and type 2 PRRSV (North American), which are defined by major genomic differences [4]. These two types of PRRSV differ both in pathogenicity [6] and virulence [5]. Therefore, the influence that pig genetics has on response to type 1 PRRSV infection still remains to be elucidated; however, a recent study conducted by Abella et al. [58] showed a favorable response of pigs with the AB genotype at the WUR SNP that were infected with a European PRRSV isolate. While these results are promising, the similarity in the host’s genetic factors that influence response to type 1 and type 2 PRRSV isolates merits further investigation.

## Conclusions

In spite of pronounced differences in viremia profiles between NVSL and KS06 PRRSV isolates, the underlying genetic factors that influence host response to infection were found to be largely the same between these two PRRSV isolates for VL, PV, and WG. Because NVSL and KS06 are diverse isolates, these results suggest that genomic selection for VL, PV, or WG during infection with one isolate will improve these traits when infected with another virus isolate. The WUR SNP, previously identified to be associated with VL and WG under infection with NVSL, was also found to be associated with all curve characteristics in the NVSL trials; but only with VL and PV in the KS06 trials, suggesting that the effect of the WUR genotype may depend on virulence of the PRRSV isolate. Infection trials with additional isolates of PRRSV are needed to confirm that genetic factors that influence host response to PRRSV infection are consistent across a range of PRRSV isolates. Genetic correlations between viremia and weight under infection at multiple time points provided insight into how the host’s genetic control of viremia and growth changes throughout the trial. Analysis of the relationship between viremia and weight gain via genome-wide association studies over the course of infection has the potential to identify additional genomic regions that could improve host response to PRRSV infection across isolates if selected for and will allow for a more comprehensive understanding of the host’s genes and genomic regions associated with response to PRRSV infection. Additional studies are currently underway as part of the PHGC and include field trials and response to vaccination and co-infection with PRRSV and PCV2b.

## Additional files

10.1186/s12711-016-0222-0 Raw viremia curves for each trial. The data provided are the plots of raw viremia across the data collection time points for all individuals from the same genetic background that have been infected with the same PRRSV isolate.
10.1186/s12711-016-0222-0 Raw means (sd) of estimated Wood’s curve parameters and proportion of animals that were classified as having cleared, rebound, or persistent serum viremia profile. The data provided are the raw averages of the Wood’s or Extended Wood’s parameters, along with the proportion of animals that were classified as cleared, rebound, or persistent.
