# Supplementary material for: Comparison of host genetic factors influencing pig response to infection with two North American isolates of porcine reproductive and respiratory syndrome virus
Source: Genet Sel Evol. 2016 Jun 20;48:43. doi: 10.1186/s12711-016-0222-0 (PMC4915112; doi:10.1186/s12711-016-0222-0)

The Kansas State University Institutional Animal Care and Use Committee approved all experimental protocols for these trials.

**Supplementary**

**Figure S1. Raw Viremia Curves for Each Trial**


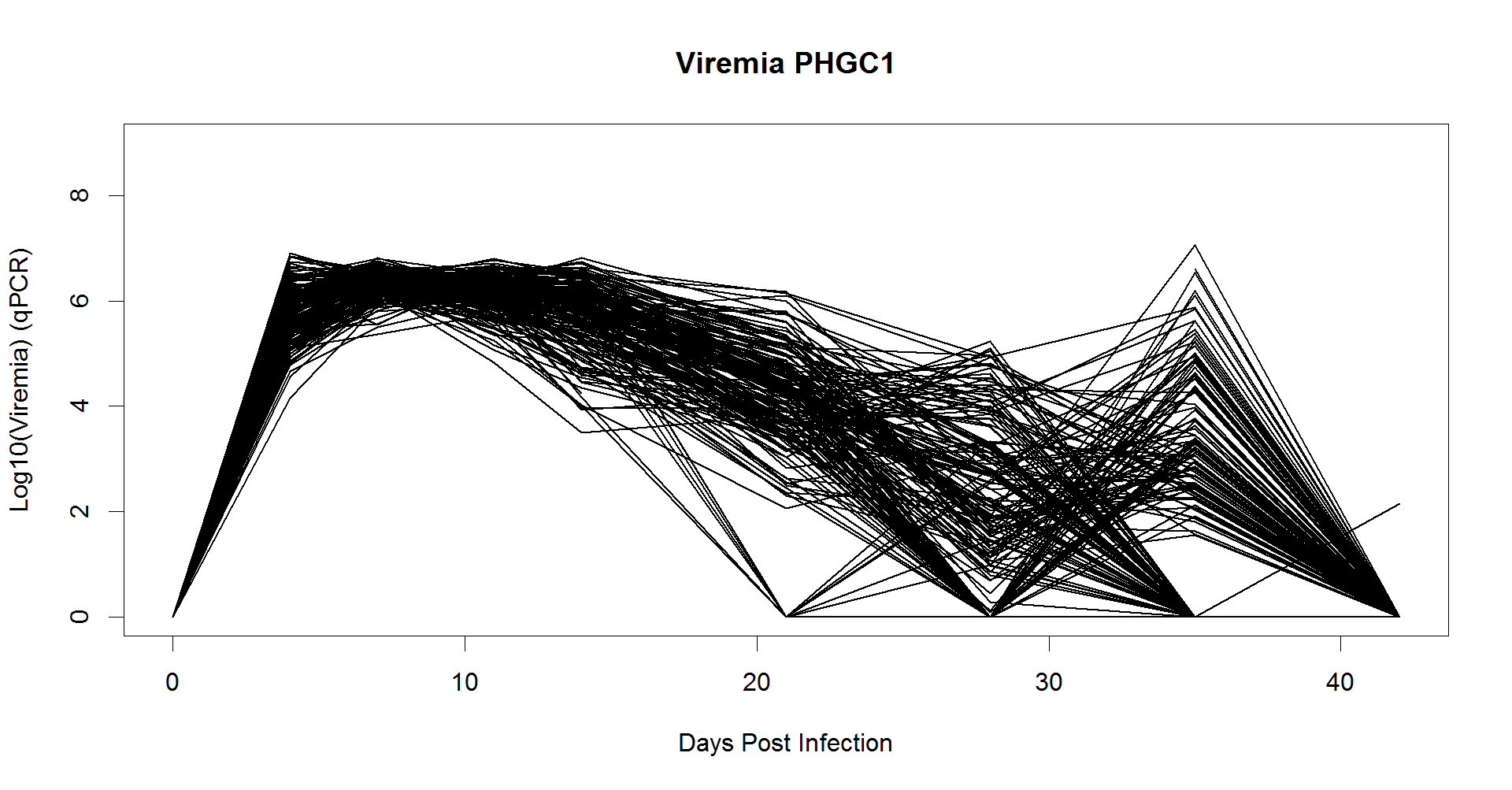


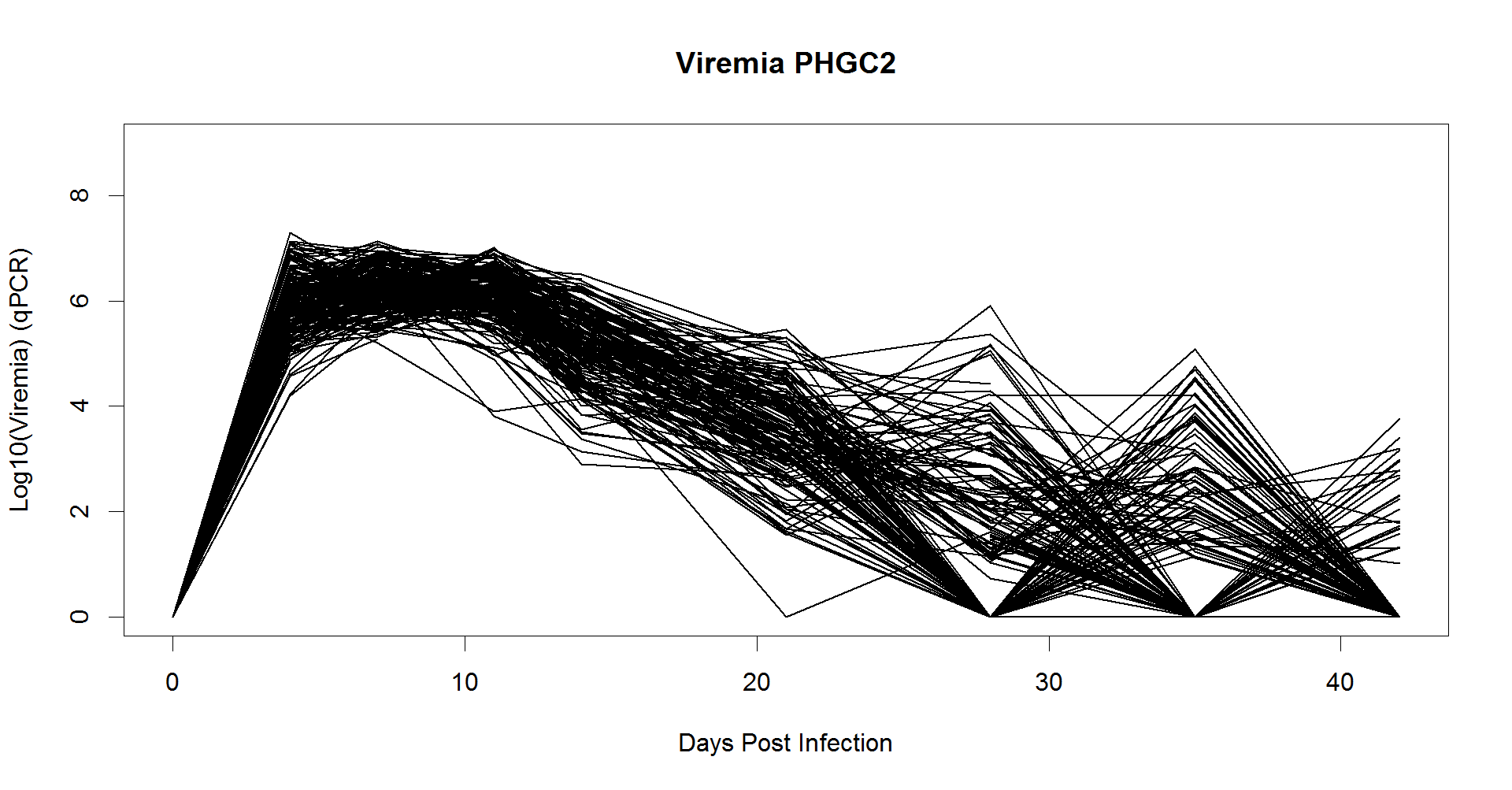

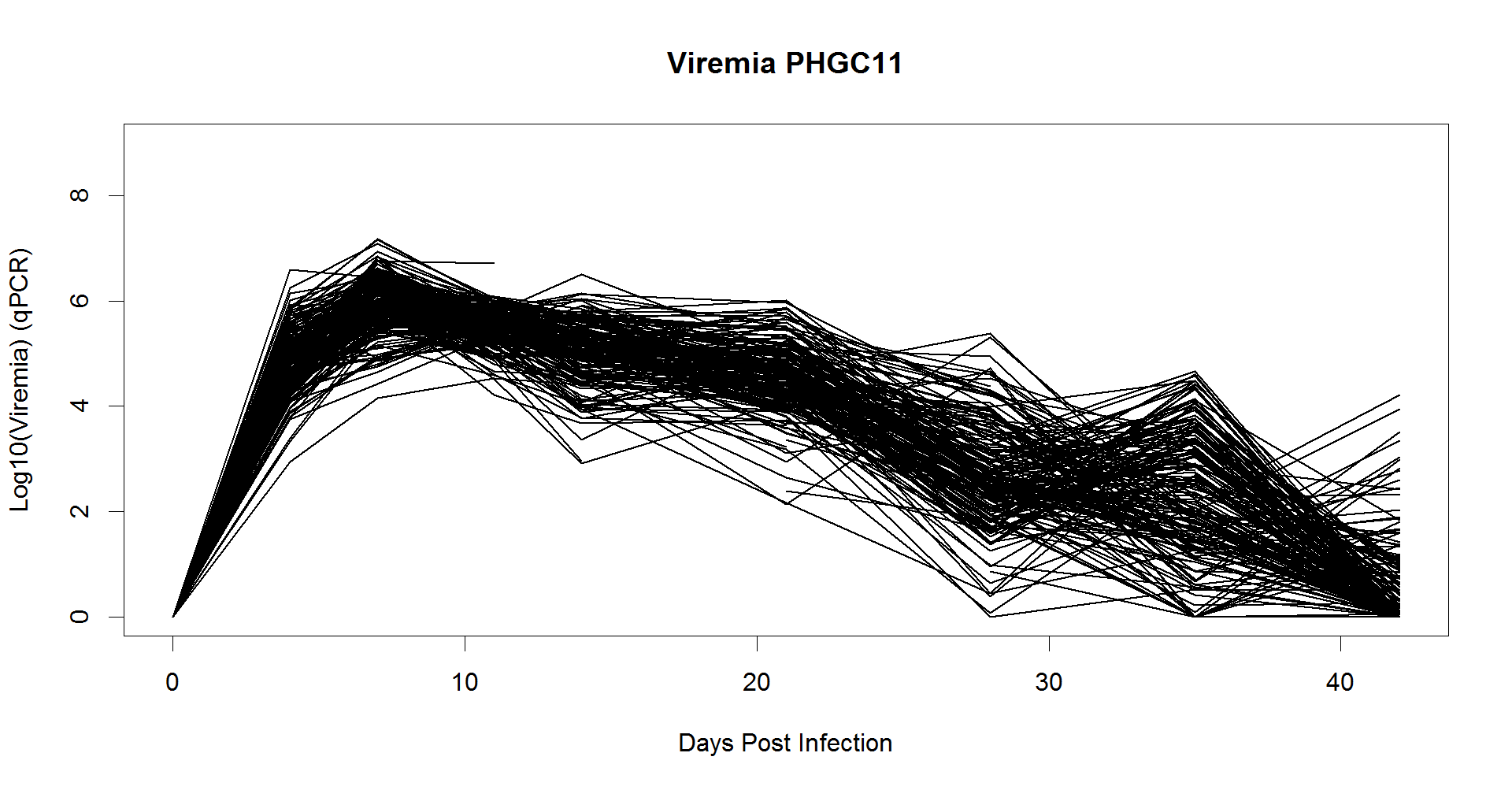


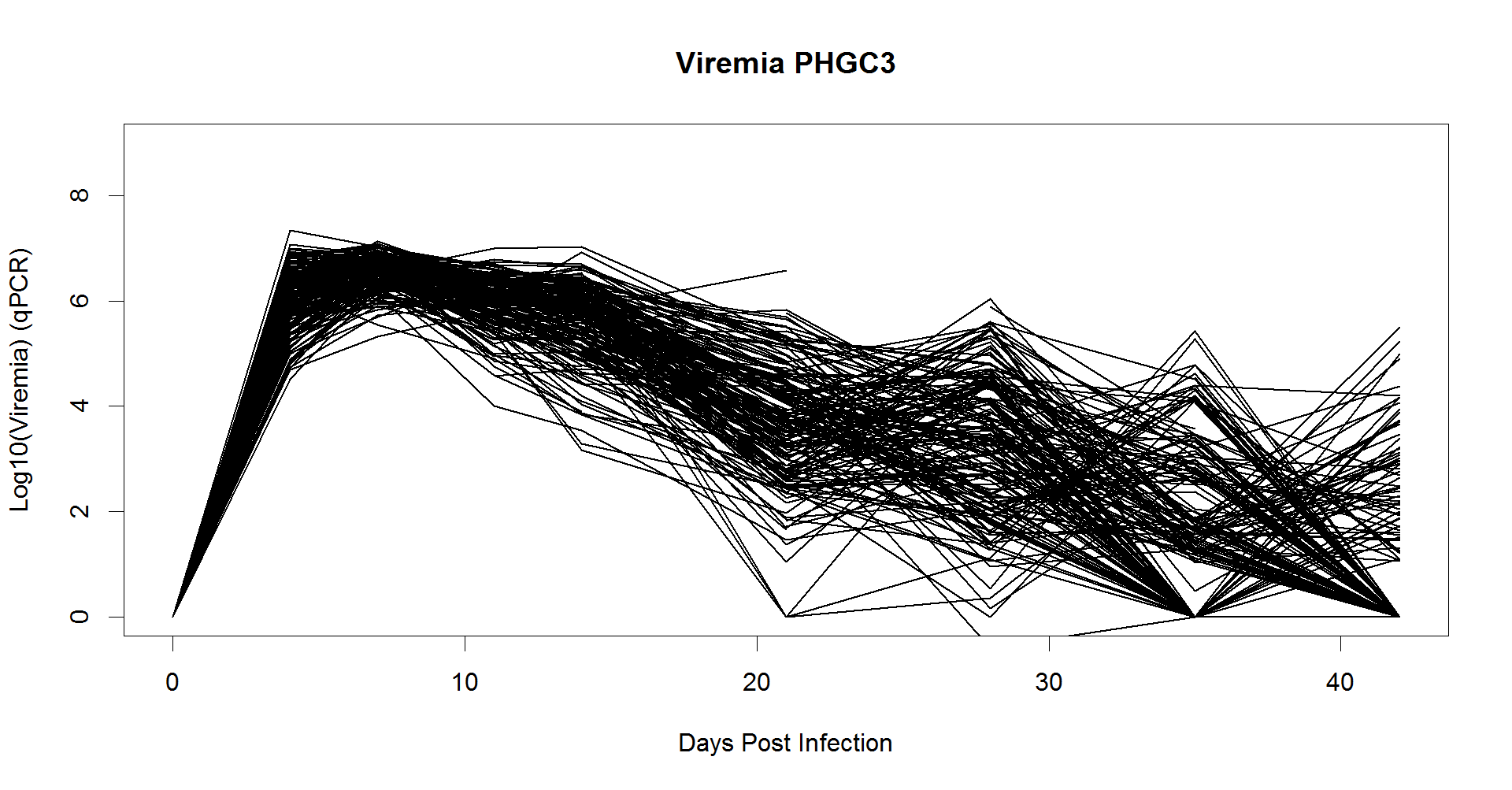


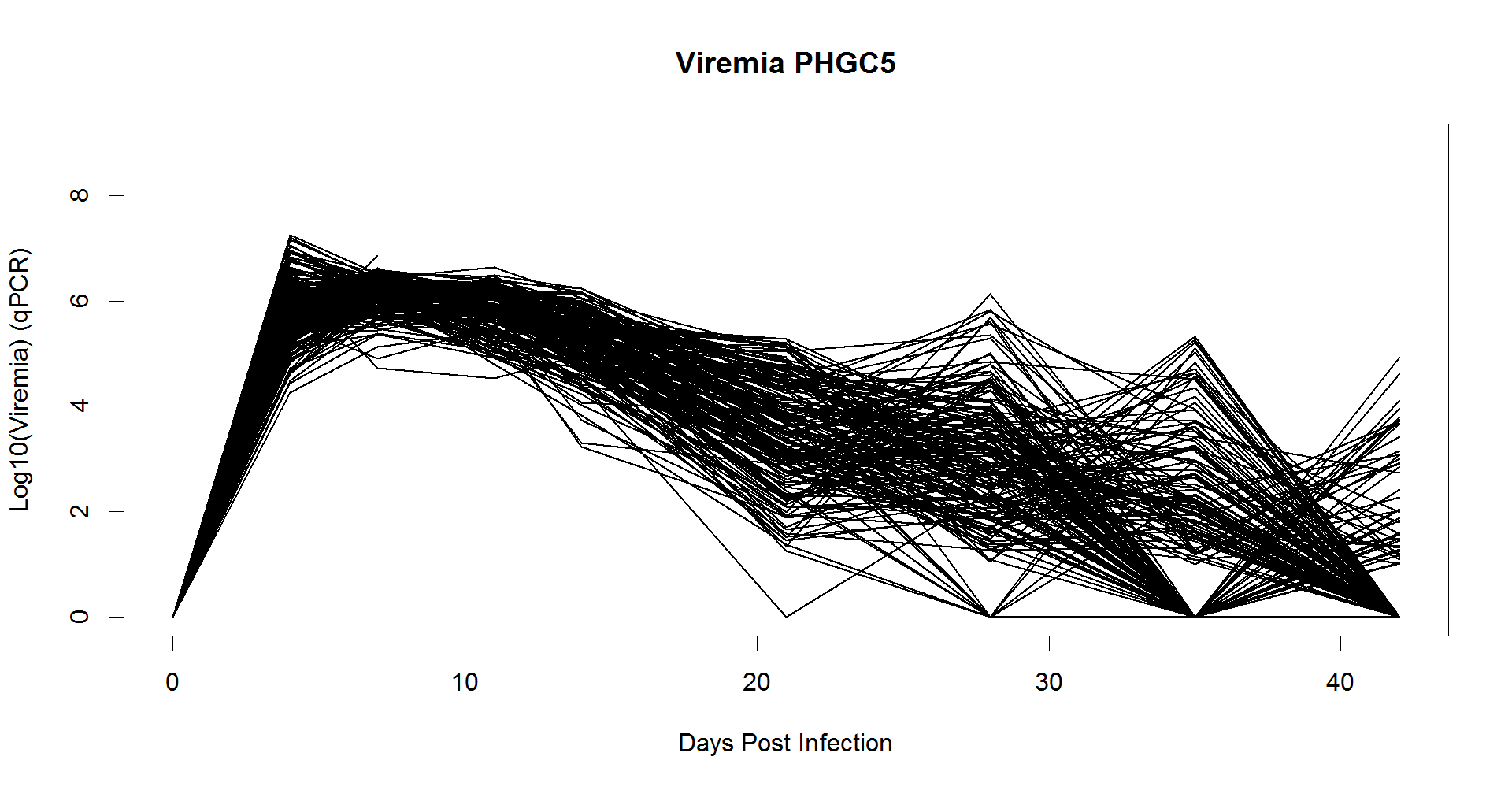

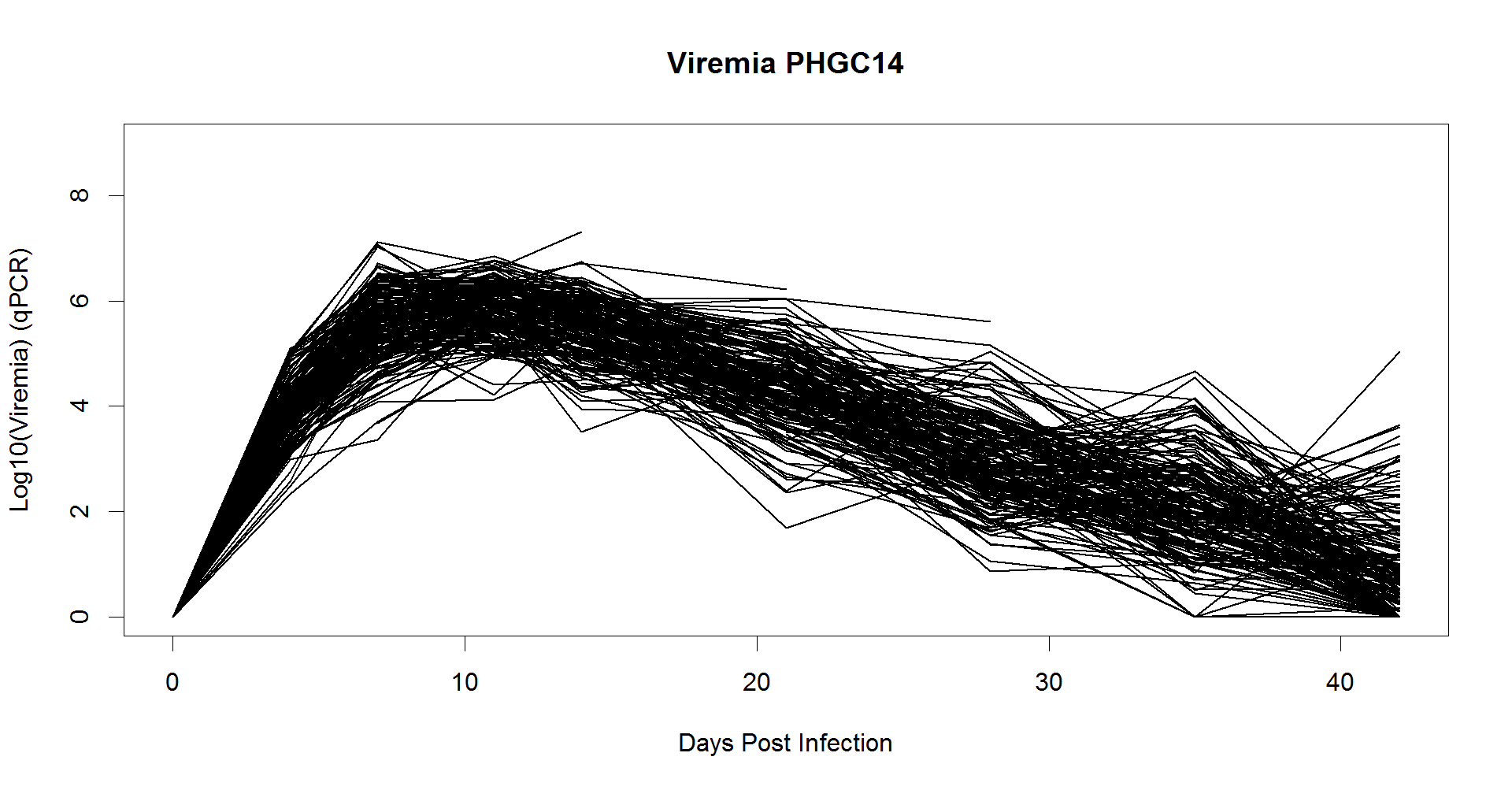


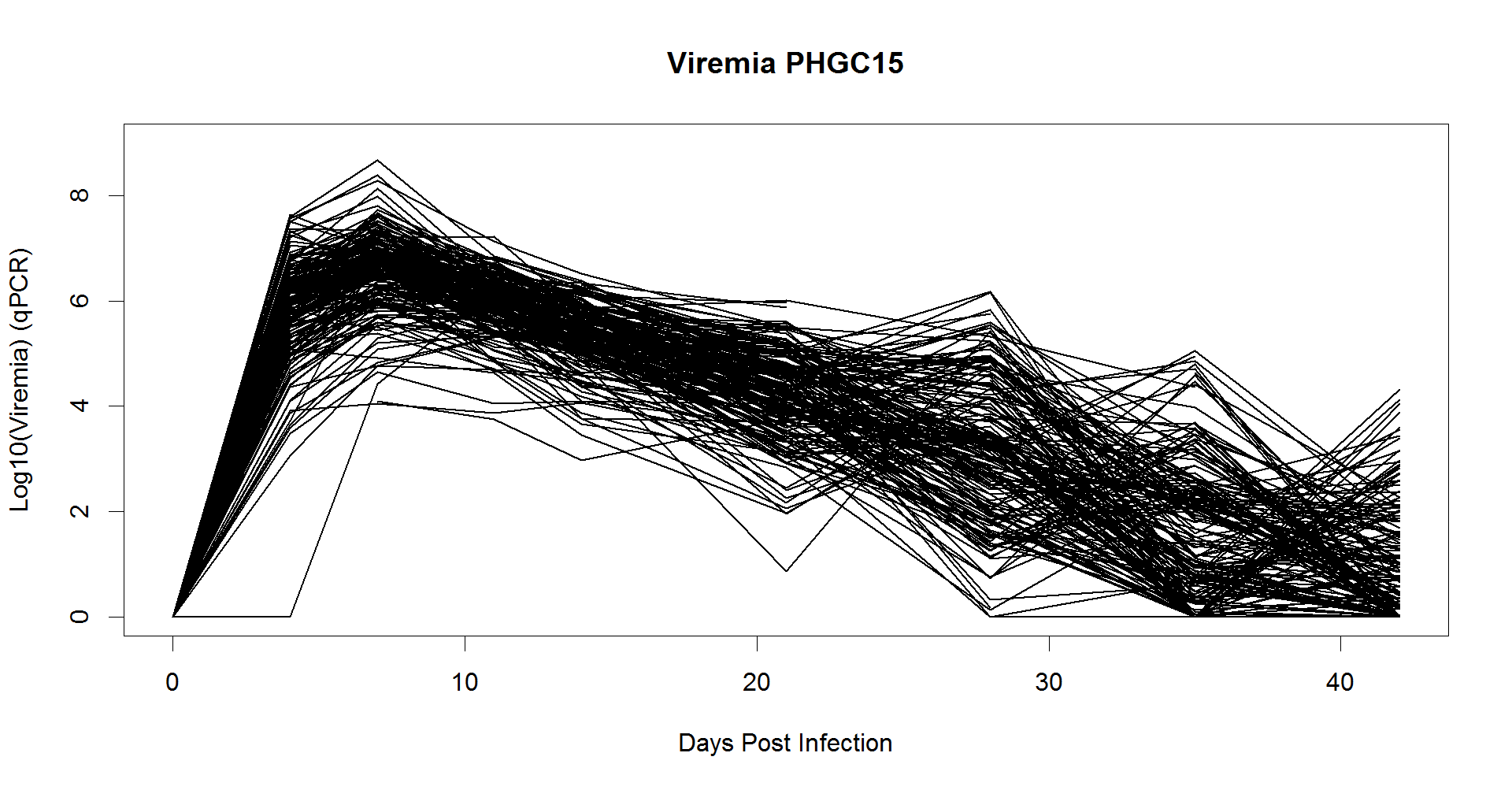

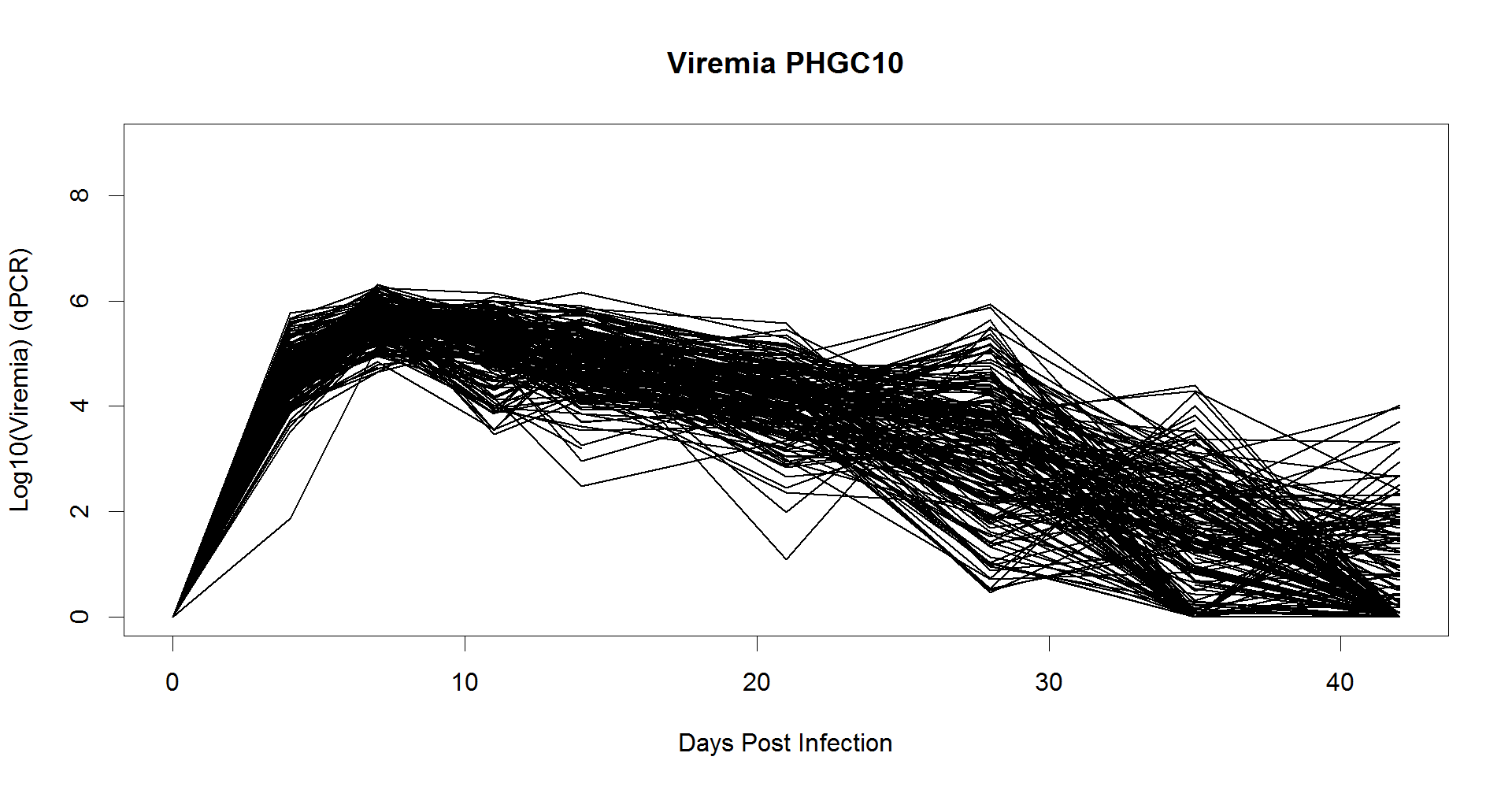


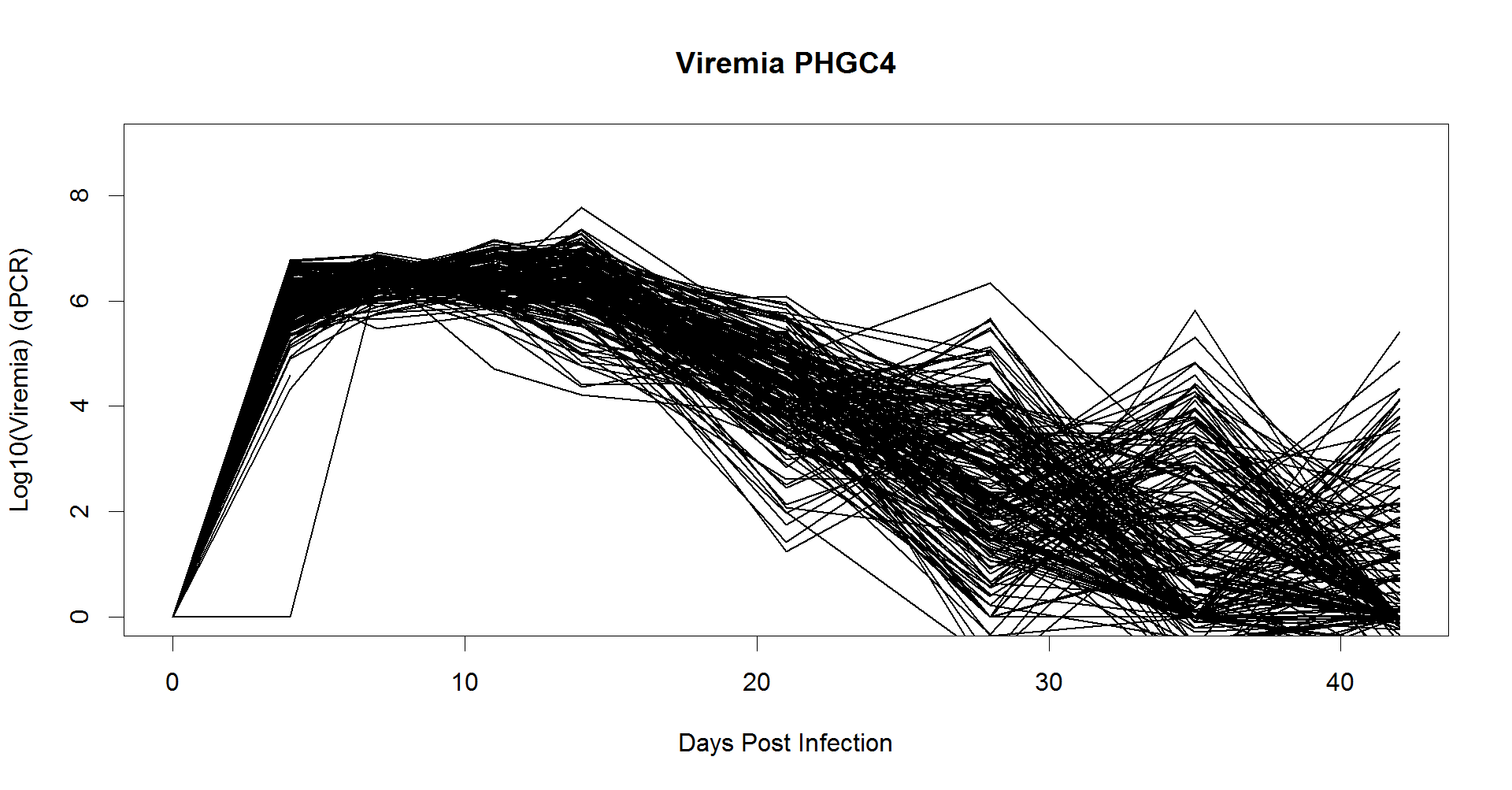

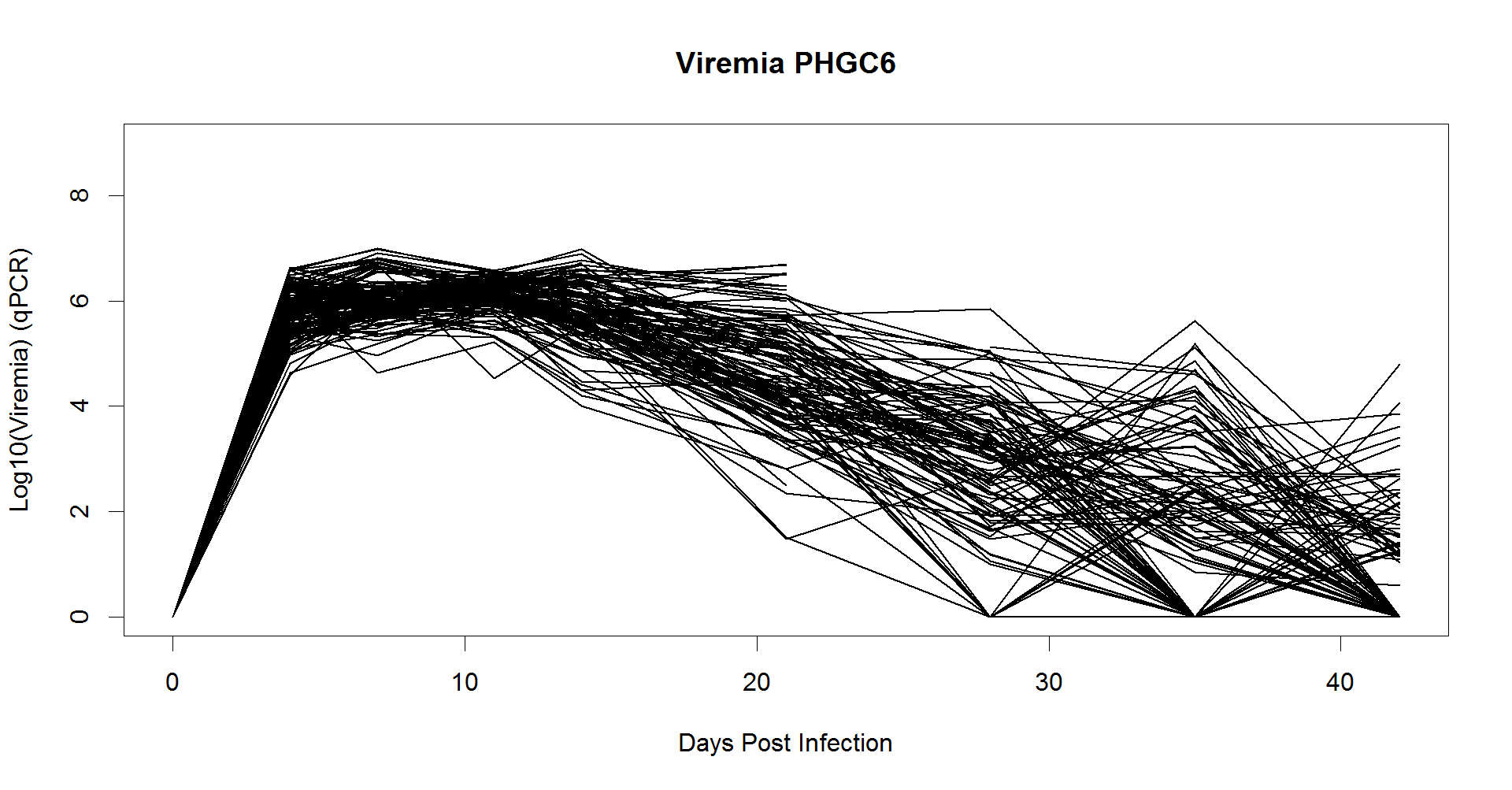


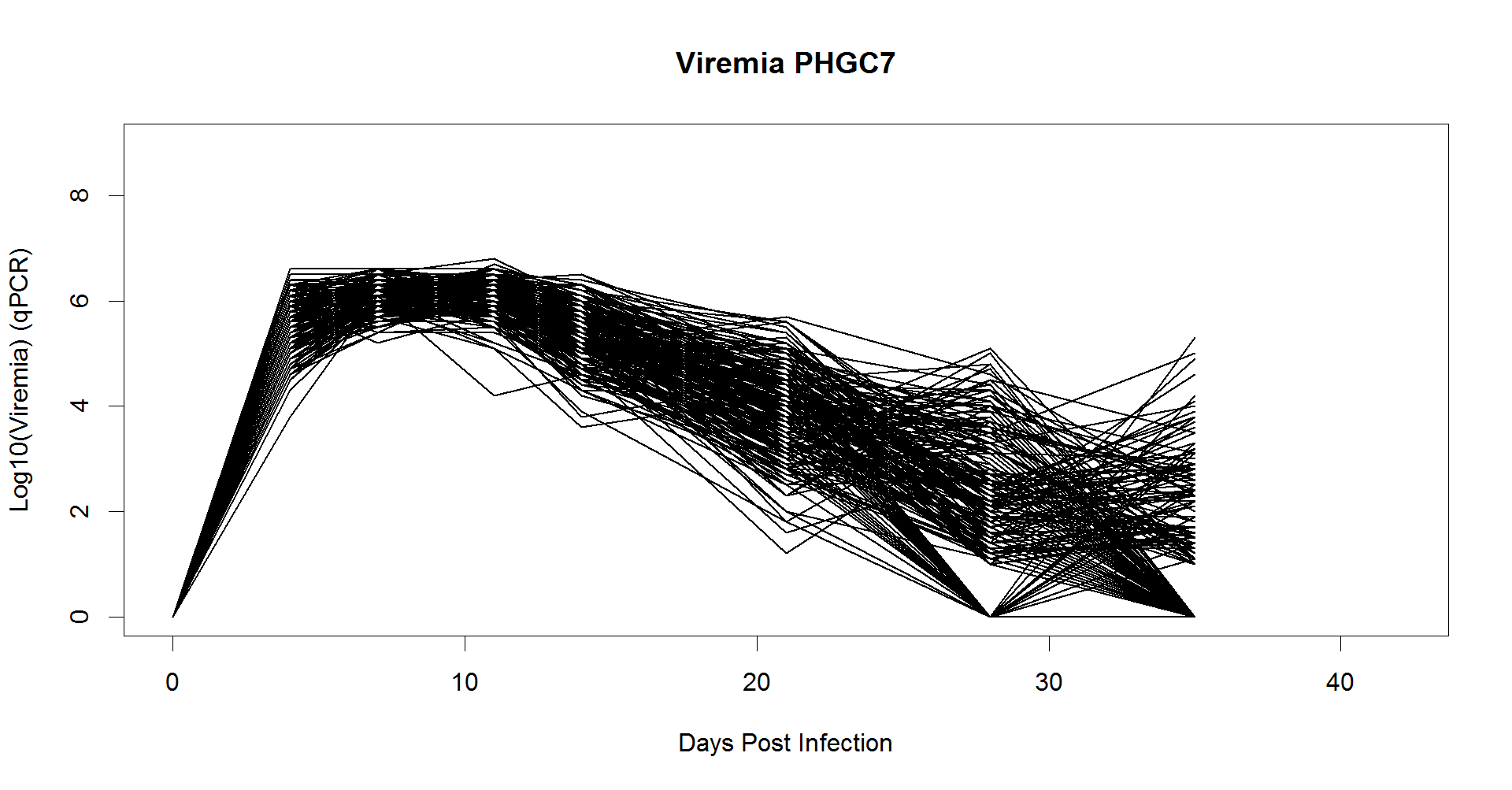

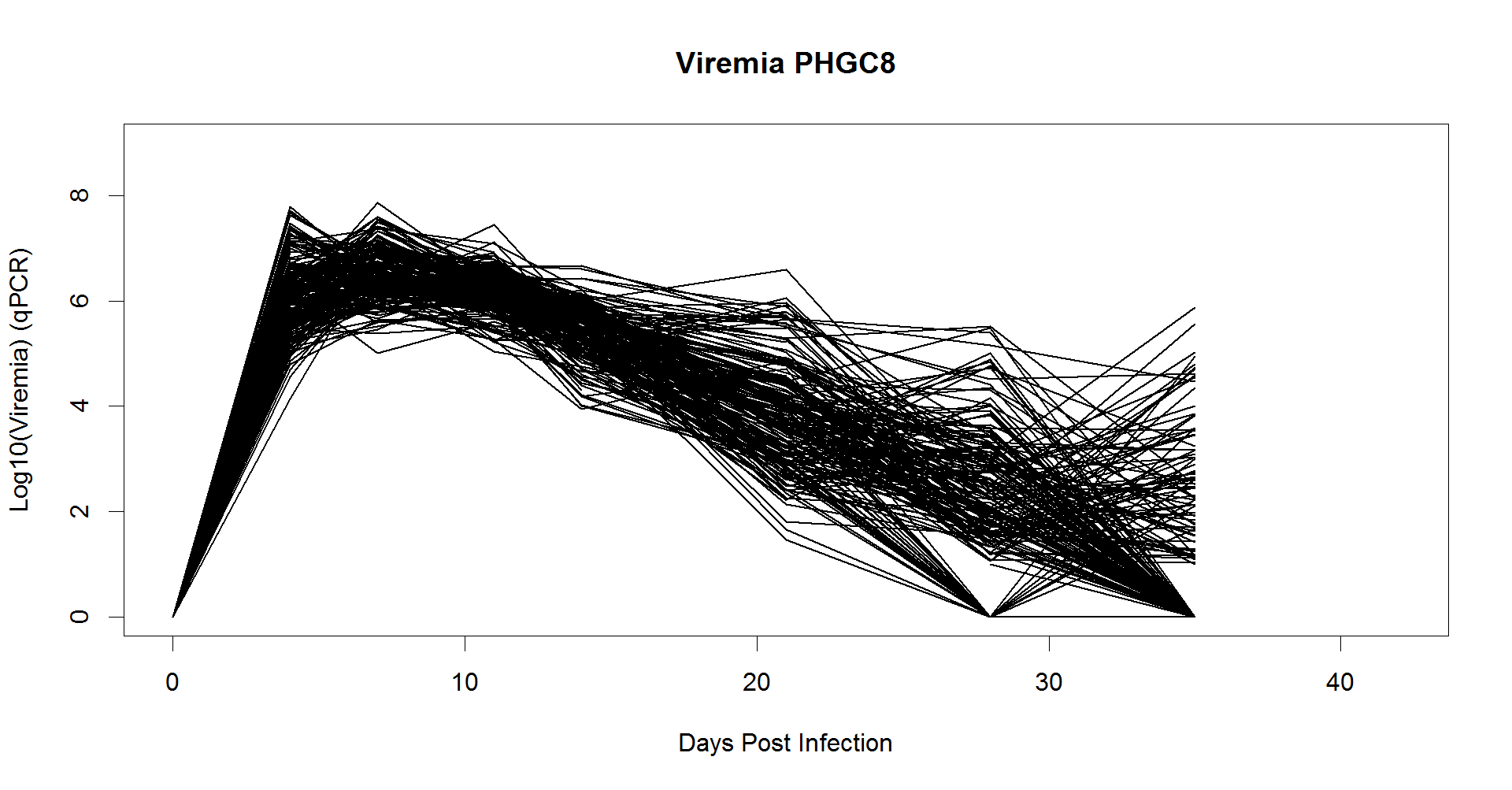


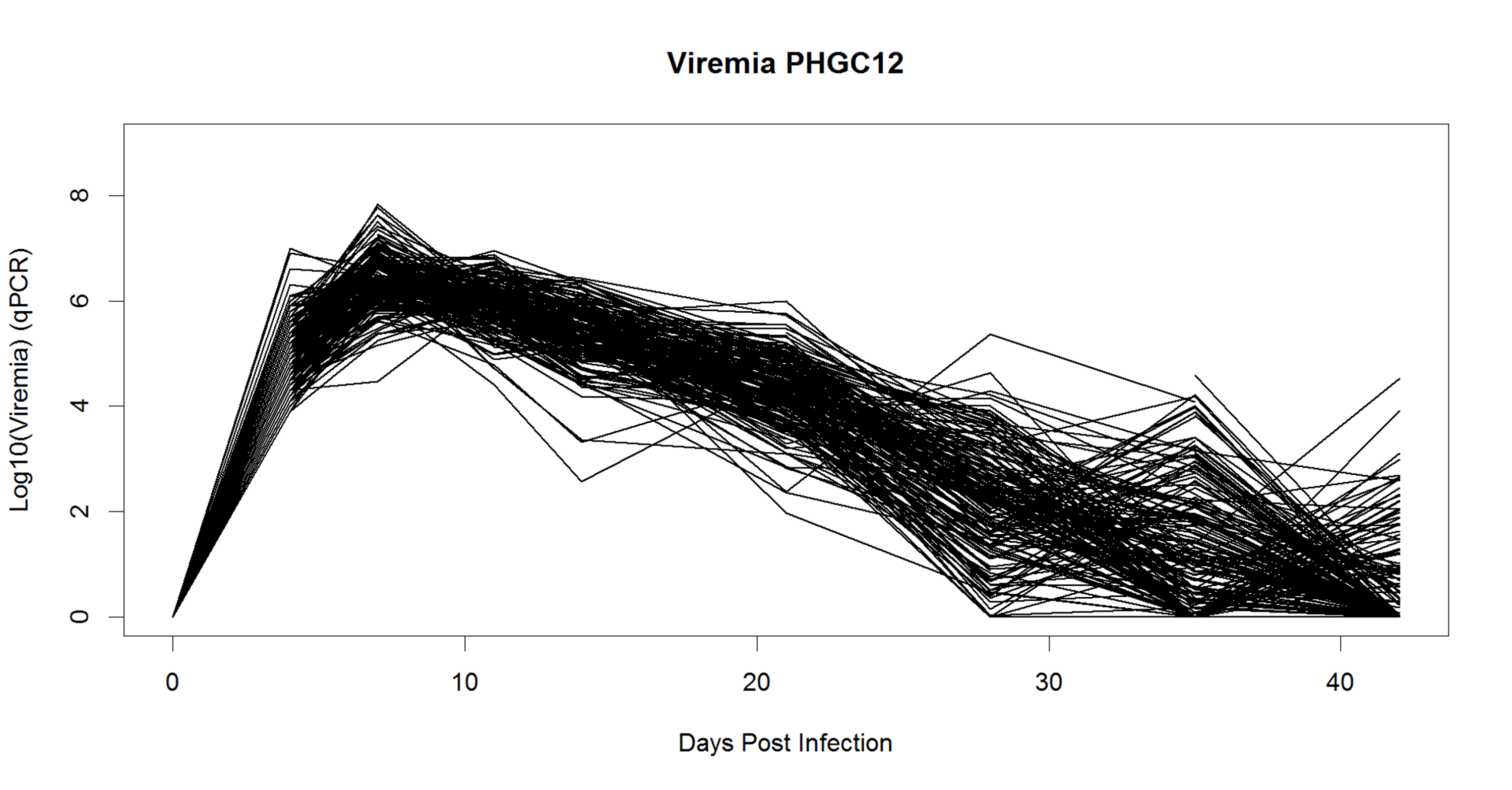

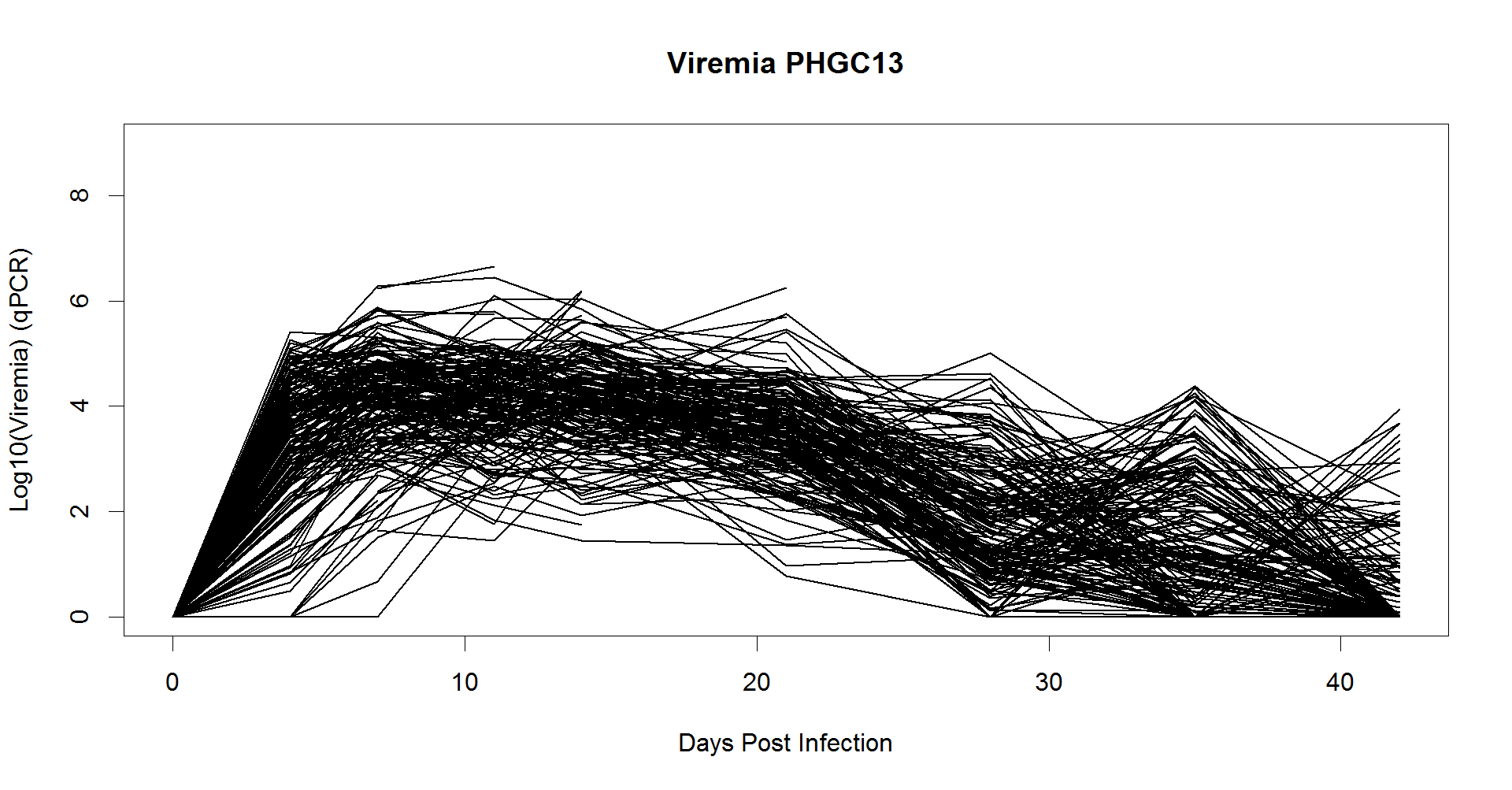

Supplement: Supplementary file 1 — 10.1186/s12711-016-0222-0 Raw viremia curves for each trial. The data provided are the plots of raw viremia across the data collection time points for all individuals from the same genetic background that have been infected with the same PRRSV isolate. [file 12711_2016_222_MOESM1_ESM.docx]
