# Supplementary material for: Comparison of host genetic factors influencing pig response to infection with two North American isolates of porcine reproductive and respiratory syndrome virus
Source: Genet Sel Evol. 2016 Jun 20;48:43. doi: 10.1186/s12711-016-0222-0 (PMC4915112; doi:10.1186/s12711-016-0222-0)
Supplement: Supplementary file 2 — 10.1186/s12711-016-0222-0 Raw means (sd) of estimated Wood’s curve parameters and proportion of animals that were classified as having cleared, rebound, or persistent serum viremia profile. The data provided are the raw averages of the Wood’s or Extended Wood’s parameters, along with the proportion of animals that were classified as cleared, rebound, or persistent. [file 12711_2016_222_MOESM2_ESM.docx]

**Table S1. Raw means (s.d.) of estimated Wood’s curve parameters and proportion of animals that were classified as having cleared, rebound, or persistent serum viremia profile**

| PRRSV Isolate | $\hat{a_{1}}$ | $\hat{b_{1}}$ | $\hat{c_{1}}$ | $\hat{a_{2}}$ | $\hat{b_{2}}$ | $\hat{c_{2}}$ | Cleared | Rebound | Persistent |
| --- | --- | --- | --- | --- | --- | --- | --- | --- | --- |
| NVSL | 3.52  (1.21) | 0.74  (0.33) | 0.10  (0.04) | 5.34  (2.64) | 5.28  (2.86) | 1.99  (1.04) | 0.44 | 0.17 | 0.40 |
| KS06 | 2.27  (1.02) | 0.86  (0.33) | 0.09  (0.03) | 4.45  (2.87) | 4.48  (2.78) | 1.64  (0.94) | 0.38 | 0.06 | 0.56 |
